# FRFNet: a fatigue-robust EEG–EMG fusion network for hand movement intention recognition

**DOI:** 10.3389/fnhum.2026.1885665

**Published:** 2026-09-11

**Authors:** Yingyu Cao, Shuoyu Bai, Zhenxi Zhao, Yongzheng He, Moxiao Fu, Xuecheng Liu, Liangfei Niu

**Affiliations:** 1 College of Mechanical Engineering, Beijing Institute of Petrochemical Technology, Beijing, China; 2 College of Mechanical Engineering, Tiangong University, Tianjin, China; 3 Xiangyu Medical Co., Ltd, Anyang, Henan, China

**Keywords:** adaptive gating, brain–computer interface, electroencephalogram, electromyogram, fatigue-robust recognition, hand movement recognition, multimodal fusion

## Abstract

**Introduction:**

Accurate movement intention recognition is essential for intelligent rehabilitation systems, wearable assistive devices, and human–machine interaction. Electroencephalography (EEG) reflects cortical motor intention and cognitive regulation, whereas surface electromyography (sEMG) provides peripheral muscle activation information related to movement execution. Although EEG–EMG fusion can provide complementary information from both the central and peripheral nervous systems, EMG signals are easily affected by muscle fatigue during long-duration or high-intensity rehabilitation tasks, resulting in amplitude attenuation, signal-to-noise ratio degradation, and modality reliability imbalance.

**Methods:**

To address this problem, this study proposes a fatigue-robust brain–muscle fusion network, termed FRFNet. The proposed framework contains a multiscale adaptive temporal convolutional network for EEG encoding, a dual-path fatigue disentanglement encoder for EMG representation, and a fatigue-aware dynamic weighted adaptive gating fusion mechanism. The EEG branch captures relatively stable movement-related cortical patterns through multiscale temporal modeling and spatial–temporal attention. The EMG branch separates fatigue-invariant action features from fatigue-sensitive degradation information, enabling implicit fatigue estimation without requiring explicit fatigue labels. The dynamic weighted adaptive gating module adjusts the contributions of EEG and EMG according to modality quality and fatigue state.

**Results:**

Experiments were conducted on a public multimodal EEG–EMG dataset involving intuitive upper-limb movement tasks. Synthetic fatigue-related EMG degradation levels from 10 to 90% were introduced for controlled robustness evaluation. Under the within-subject protocol, FRFNet achieved 60.0% accuracy at 90% degradation, exceeding E2FNet, DCA Fusion, and DMEFNet-adapted by 12.7, 15.2, and 11.7 percentage points, respectively. Under strict leave-one-subject-out evaluation, FRFNet achieved 57.0% accuracy at the same level and maintained the highest overall performance among the six evaluated methods. The deployment inference path contained 1.166 million parameters and achieved a mean single-trial forward latency of 5.321 ± 0.934 ms.

**Conclusion:**

The results indicate that degradation-aware adaptive fusion can suppress unreliable EMG features and improve EEG–EMG movement decoding under progressive EMG degradation. FRFNet therefore provides a robust multimodal decoding framework for fatigue-aware rehabilitation and adaptive human–machine interaction under the evaluated synthetic EMG degradation conditions.

## 1 Introduction

In recent years, intelligent rehabilitation systems, wearable assistive devices, and human–machine interaction technologies have developed rapidly, providing new technical approaches for motor function assessment, movement assistance, and rehabilitation training. As a core component of these systems, movement intention recognition aims to infer the user’s intended action from physiological signals generated during motor planning and movement execution. Accurate and robust movement intention recognition can help rehabilitation robots and assistive devices provide timely feedback and adaptive control, which is of great significance for patients with stroke, spinal cord injury, neuromuscular impairment, and limb motor dysfunction.

Among different biological signals, electroencephalography (EEG) and surface electromyography (sEMG) are two representative modalities widely used in movement intention recognition. EEG reflects neural activities related to motor intention, cognitive preparation, and sensorimotor rhythm modulation, and can provide information from the central nervous system before or during movement execution (Pfurtscheller and Lopes da Silva, 1999). In contrast, sEMG directly reflects peripheral muscle activation and is closely associated with actual movement execution. Therefore, EEG and EMG describe the human motor control process from different physiological levels. The former emphasizes cortical motor intention, while the latter reflects muscle response and execution state. The fusion of EEG and EMG can provide complementary information from the central and peripheral nervous systems, thereby improving the reliability and accuracy of movement intention recognition compared with single-modality methods (Yang et al., 2022; Tang et al., 2025; Jiang et al., 2024).

EEG–EMG fusion has been increasingly applied in rehabilitation robots, hybrid brain–computer interfaces, exoskeleton control, and human–machine interaction systems. Non-invasive BCI technologies have been widely explored for communication, assistive control, and neurorehabilitation, and previous studies have shown their potential in robotic device control and stroke rehabilitation training (Pfurtscheller and Neuper, 2001; Tonin and Millán, 2021; Baniqued et al., 2021; Xu et al., 2021). In rehabilitation scenarios, patients may have weak or unstable muscle activation. EEG can provide intention-related information from the central nervous system, whereas EMG provides direct evidence of residual muscle activity. Clinical studies have also shown that BCI-based training, motor imagery rehabilitation, and BCI-controlled electrical stimulation can improve upper- or lower-limb motor function after stroke (Kim et al., 2016; Liao et al., 2023; Luo, 2024; Ming et al., 2025; Shen et al., 2025). Therefore, EEG–EMG fusion is suitable for movement intention recognition under complex rehabilitation conditions.

For EEG decoding, traditional spatial filtering and spectral feature extraction methods remain important foundations, including CSP, FBCSP, spatio-spectral filters, and regularized CSP variants (Ramoser et al., 2000; Ang et al., 2008; Herman et al., 2008; Lemm et al., 2005; Dornhege et al., 2006; Thomas et al., 2009; Lotte and Guan, 2010). Functional connectivity analysis has also been used to characterize interactions among brain regions during motor imagery tasks (Leeuwis et al., 2021). With the development of deep learning, Deep ConvNet, EEGNet, FBCNet, EEG Conformer, and LMDA-Net have improved EEG feature extraction through convolution, multi-view spectral representation, self-attention, and multi-dimensional attention mechanisms (Schirrmeister et al., 2017; Lawhern et al., 2018; Mane et al., 2021; Song et al., 2023; Miao et al., 2023). Artifact removal methods such as IC-U-Net can further improve EEG preprocessing under noisy conditions (Chuang et al., 2022). However, EEG alone still has limitations in fine-grained hand movement recognition because of its low spatial resolution, low signal-to-noise ratio, and inter-subject variability.

Compared with EEG, surface EMG is more directly related to muscle activation and movement execution, but it is easily influenced by fatigue, electrode shift, skin impedance variation, and subject-specific activation patterns. During repeated grasping or long-duration rehabilitation exercises, muscle fatigue changes the statistical properties of EMG signals, including amplitude attenuation, reduced muscle fiber conduction velocity, spectral shift toward lower frequencies, and signal-to-noise ratio degradation (Cifrek et al., 2009; Sun et al., 2022). Classical EMG feature extraction studies have shown that time-domain, frequency-domain, and higher-order statistical features are useful for myoelectric pattern recognition, but these handcrafted features may be sensitive to amplitude variation and noise (Nazarpour et al., 2007; Phinyomark et al., 2012; Côté-Allard et al., 2019).

EEG–EMG fusion combines cortical intention information and peripheral execution information. Tryon and Trejos explored EEG–EMG fusion for dynamic task weight classification and CNN-based fusion (Tryon and Trejos, 2021a; Tryon and Trejos, 2021b). Yang et al. proposed a graph convolutional method based on EEG–sEMG functional connectivity for robust hand motion recognition under fatigue simulation (Yang et al., 2022). Shi et al. introduced DMEFNet with dense co-attention for multimodal human–exoskeleton movement prediction (Shi et al., 2022). Tang et al. proposed attention-based multiscale parallel convolution and dual-stream spectral fusion frameworks for EEG–sEMG decoding (Tang et al., 2024; Tang et al., 2025). Jiang et al. proposed E2FNet with multiscale convolution and cross-modal feature enhancement for hand movement intention recognition (Jiang et al., 2024). These studies demonstrate the effectiveness of EEG–EMG fusion in feature extraction and cross-modal interaction. However, their fusion relationships are mainly learned from action-classification objectives and do not explicitly represent the sample-dependent degradation state of EMG. Consequently, when EMG becomes unreliable under progressive fatigue, the degraded modality may still retain excessive influence on the fused representation. The methodological objective of FRFNet is therefore not to introduce temporal convolution or attention as new operations, but to establish a fatigue-specific closed loop that separates relatively stable action information from degradation-sensitive EMG information, estimates the degradation state without explicit fatigue labels, and uses this state together with modality confidence to regulate EEG–EMG contributions.

To achieve this objective, this study proposes a fatigue-robust EEG–EMG fusion network, termed FRFNet. Its core design consists of two coupled components. First, the dual-path fatigue decoupling encoder employs pathway-specific normalization and feature usage to produce a relatively fatigue-invariant EMG action representation and a fatigue-sensitive degradation estimate. Second, the dynamic weighted adaptive gating module jointly uses sample-dependent gating information, modality confidence, and the estimated degradation state to reallocate EEG and EMG contributions. MS-ATCN and enhanced co-attention are used as supporting modules for stable EEG representation and cross-modal interaction, respectively, rather than being claimed as standalone methodological novelties.

The experiments were conducted on the public multimodal EEG–EMG dataset released by Jeong et al. (2020), which provides synchronized EEG, EMG, and EOG recordings during intuitive upper-limb movement tasks. Related public EEG resources have also supported reproducible BCI research and multimodal decoding studies (Cho et al., 2017; Jeong et al., 2020). By introducing synthetic fatigue levels from 10 to 90%, this study systematically evaluates model robustness under progressive EMG degradation.

The main contributions of this paper are summarized as follows:

(1) A fatigue-specific EEG–EMG recognition framework is developed to address the progressive reliability imbalance caused by EMG degradation. Unlike conventional fusion methods that learn a fixed or implicit modality relationship, FRFNet explicitly introduces degradation-state information into both EMG representation and multimodal fusion.
(2) A dual-path fatigue decoupling strategy is designed for EMG representation. Through pathway-specific preprocessing and feature usage, the fatigue-invariant path suppresses global amplitude variation and extracts action-discriminative information, whereas the fatigue-sensitive path preserves degradation-related energy variation. The former is used for movement classification, while the latter provides a label-free fatigue-related state signal for subsequent fusion.
(3) A fatigue-conditioned dynamic weighted adaptive gating mechanism is proposed. Instead of generating fusion weights only from multimodal features, DW-AGF jointly incorporates sample-dependent gating information, modality confidence, and the fatigue estimate produced by the EMG encoder. This coupling forms a closed loop from EMG degradation detection to reliability-aware modality reallocation.
(4) Through robust training and fatigue-aware adaptive fusion, FRFNet maintains a slower performance degradation trend across the 10–90% synthetic fatigue range. The evaluation includes both within-subject nested five-fold cross-validation and strict leave-one-subject-out testing, together with subject-level statistical analysis, component-level ablation, and hyperparameter sensitivity experiments. These results provide evidence for both robustness to synthetic fatigue-related EMG degradation and improved unseen-subject generalization relative to the evaluated baselines.

The rest of this paper is organized as follows. Section 2 introduces the proposed FRFNet framework, including the EEG encoder, EMG dual-path fatigue disentanglement encoder, and fatigue-aware dynamic weighted fusion mechanism. Section 3 describes the dataset, fatigue simulation strategy, preprocessing procedure, experimental settings, and evaluation metrics. Section 4 presents the experimental results and comparison with existing methods. Section 5 discusses the effectiveness of the proposed modules and analyzes the influence of fatigue-aware fusion. Section 6 concludes the paper and outlines future work.

## 2 Methods

### 2.1 Overall architecture of FRFNet

**Figure 1:**
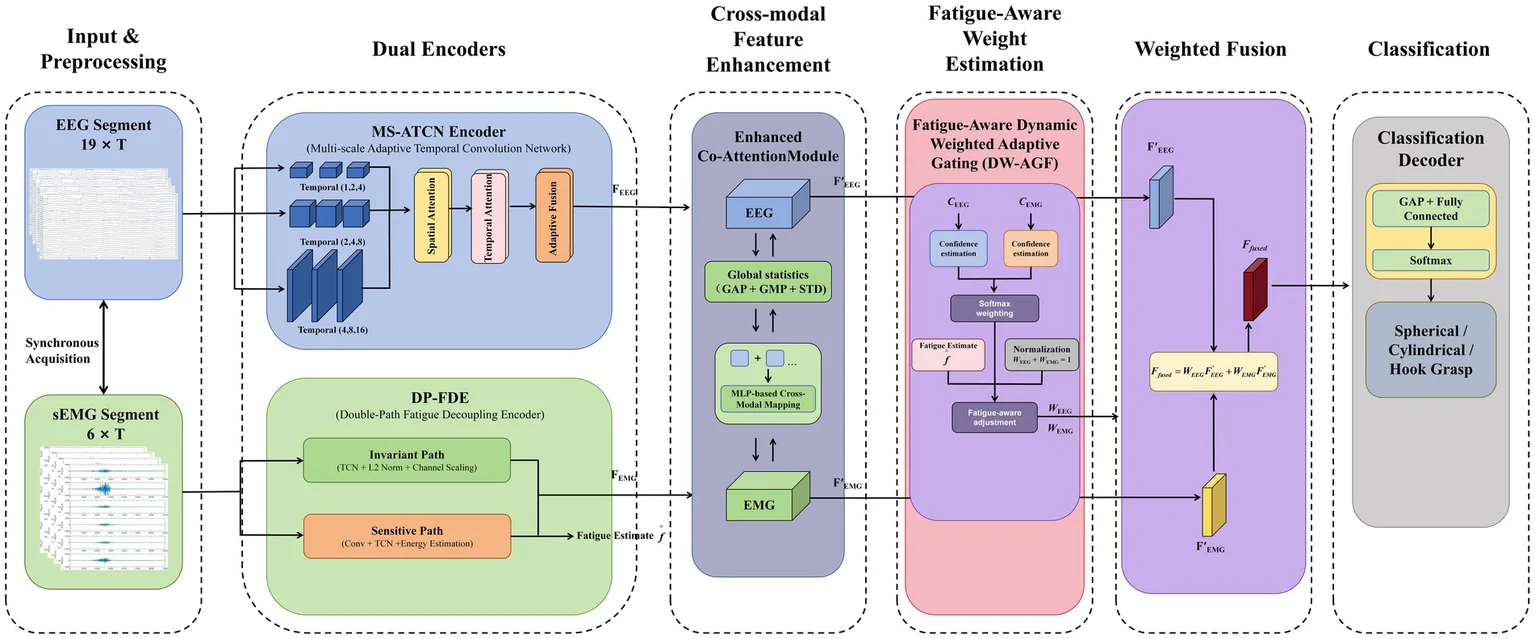
Overall architecture of FRFNet.

The proposed FRFNet adopts a dual-modality parallel encoding and adaptive fusion architecture. It contains four major components: an EEG encoder based on multiscale adaptive temporal convolution, an EMG encoder based on dual-path fatigue disentanglement, an enhanced co-attention interaction module, and a fatigue-aware dynamic weighted adaptive gating classifier. The overall architecture of FRFNet is shown in Figure 1.

Let the EEG input be denoted as follows (Equation 1):


$$X_{\mathrm{EEG}}\in {\mathbb{R}}^{B\times C_{E}\times T}$$ (1)

Let the EMG input be denoted as follows (Equation 2):


$$X_{\mathrm{EMG}}\in {\mathbb{R}}^{B\times C_{M}\times T}$$ (2)

where B is the batch size, $C_{E}$ is the number of EEG channels, $C_{M}$ is the number of EMG channels, and T is the number of temporal samples. The goal of the network is to learn a mapping from synchronized EEG–EMG signals to the hand movement class label y .

The EEG branch is designed to extract stable cognitive and movement intention features. EEG signals contain sensorimotor rhythm changes and cortical activation patterns related to movement preparation and execution. However, these patterns may appear at different time scales and are distributed across different channels. Therefore, the EEG encoder uses parallel temporal convolution branches with different dilation rates and attention mechanisms to capture multiscale temporal dependencies and spatial–temporal discriminative patterns.

The EMG branch is designed to handle fatigue-induced degradation. Instead of using a single encoder for all EMG information, the proposed DP-FDE divides EMG representation into two paths. The fatigue-invariant path uses normalized EMG input to reduce the influence of amplitude attenuation and extract stable action-related features. The fatigue-sensitive path preserves amplitude-related information and estimates fatigue degree based on signal energy decay. This design reduces the mixing of action features and fatigue degradation factors.

After the EEG and EMG features are extracted, an enhanced co-attention module performs bidirectional interaction between the two modalities. The co-attention module allows EEG features to be modulated by EMG global statistics and EMG features to be modulated by EEG global statistics, thereby improving cross-modal feature consistency. Then, the DW-AGF module calculates modality confidence and fatigue-aware weights. The final fused feature is obtained by weighted summation of EEG and EMG features and is passed to a global average pooling layer and a fully connected classifier for action prediction.

### 2.2 EEG encoder: multiscale adaptive temporal convolutional network

#### 2.2.1 Multiscale temporal branches

EEG signals are non-stationary and contain information at different temporal scales. Short temporal windows may capture transient event-related responses, medium windows may capture sensorimotor rhythm modulation, and longer windows may capture sustained movement-related neural activity. To model these characteristics, the EEG encoder contains three parallel temporal convolution branches with different dilation rates.

For the i -th branch, the adaptive temporal convolution block is defined as follows (Equation 3):


$$F_{i}=\text{GELU}(\mathrm{BN}(\text{Conv}1D_{d_{i}}(X_{\mathrm{EEG}})))⊙\alpha_{i}$$ (3)

where $\text{Conv}1D_{d_{i}}$ denotes a one-dimensional temporal convolution with dilation rate $d_{i}$ , BN denotes batch normalization, GELU is the activation function, $\alpha_{i}$ is a channel-wise adaptive scaling factor, and ⊙ denotes element-wise multiplication. In this study, three branches with small, medium, and large dilation rates are used to capture local, intermediate, and long-range temporal dependencies.

The adaptive scaling factor is generated from global statistical information of the input feature (Equation 4):


$$\alpha_{i}=\sigma (\mathrm{MLP}(\mathrm{GAP}(F_{i})))$$ (4)

where GAP denotes global average pooling along the temporal dimension, MLP denotes a multilayer perceptron, and σ(·) denotes the sigmoid function. This design allows the network to adaptively recalibrate channel responses according to the sample-specific EEG distribution.

#### 2.2.2 Spatial–temporal attention mechanism

Movement-related EEG information is not uniformly distributed across all channels and time points. Channels near the motor cortex may contain stronger discriminative information, and specific temporal segments may be more relevant to movement classification. Therefore, spatial and temporal attention mechanisms are introduced after multiscale temporal feature extraction.

The spatial attention weight is computed as follows (Equation 5):


$$A_{s}=\sigma (\text{Conv}1D({\mathrm{GAP}}_{t}(F_{E})))$$ (5)

where $F_{E}$ denotes the multiscale EEG feature, ${\mathrm{GAP}}_{t}$ denotes global average pooling along the temporal dimension, and $A_{s}$ represents the channel attention weight. The spatially enhanced EEG feature is calculated as follows (Equation 6):


$$F_{E}^{s}=F_{E}⊙A_{s}$$ (6)

The temporal attention weight is computed as follows (Equation 7):


$$A_{t}=\sigma (\text{Conv}1D({\mathrm{GAP}}_{c}(F_{E}^{s})))$$ (7)

where ${\mathrm{GAP}}_{c}$ denotes global average pooling along the channel dimension. The final EEG representation is obtained as follows (Equation 8):


$$Z_{E}=F_{E}^{s}⊙A_{t}$$ (8)

Through spatial–temporal attention, the EEG encoder can focus on both important brain regions and discriminative time segments. This is beneficial for extracting stable movement intention features under noisy EEG conditions.

#### 2.2.3 Learnable multiscale fusion

Let the outputs of the three EEG temporal branches be $F_{1}$ , $F_{2}$ , and $F_{3}$ . A simple concatenation of multiscale features may introduce redundant information, while fixed averaging cannot adapt to sample-specific temporal patterns. Therefore, a learnable weighted fusion strategy is used.

First, each branch feature is compressed into a global statistical vector (Equation 9):


$$s_{i}=\mathrm{GAP}(F_{i}),\;\;i=1,2,3$$ (9)

Then, the three statistical vectors are concatenated and fed into an MLP followed by a softmax function (Equation 10):


$$\left[w_{1},w_{2},w_{3}]=\text{Softmax}(\mathrm{MLP}([s_{1};s_{2};s_{3}])\right)$$ (10)

The fused multiscale EEG feature is calculated as follows (Equation 11):


$$F_{E}=w_{1}F_{1}+w_{2}F_{2}+w_{3}F_{3}$$ (11)

This strategy allows the EEG encoder to adaptively select the most useful temporal scale for each sample. For example, when short-term transient changes are more informative, the branch with a smaller receptive field may receive a higher weight. When long-range temporal dependencies are more important, the branch with a larger dilation rate may contribute more.

### 2.3 EMG encoder: dual-path fatigue disentanglement modeling

#### 2.3.1 Motivation

Surface EMG signals are strongly related to muscle activation and often provide highly discriminative information for hand movement recognition. However, EMG is also sensitive to muscle fatigue. During fatigue accumulation, the EMG signal may show amplitude attenuation, changes in frequency components, and increased noise (Cifrek et al., 2009; Sun et al., 2022). If all EMG information is encoded through a single path, action-discriminative features and fatigue-induced degradation factors may be entangled. This entanglement can reduce feature stability and mislead the fusion module.

**Figure 2:**
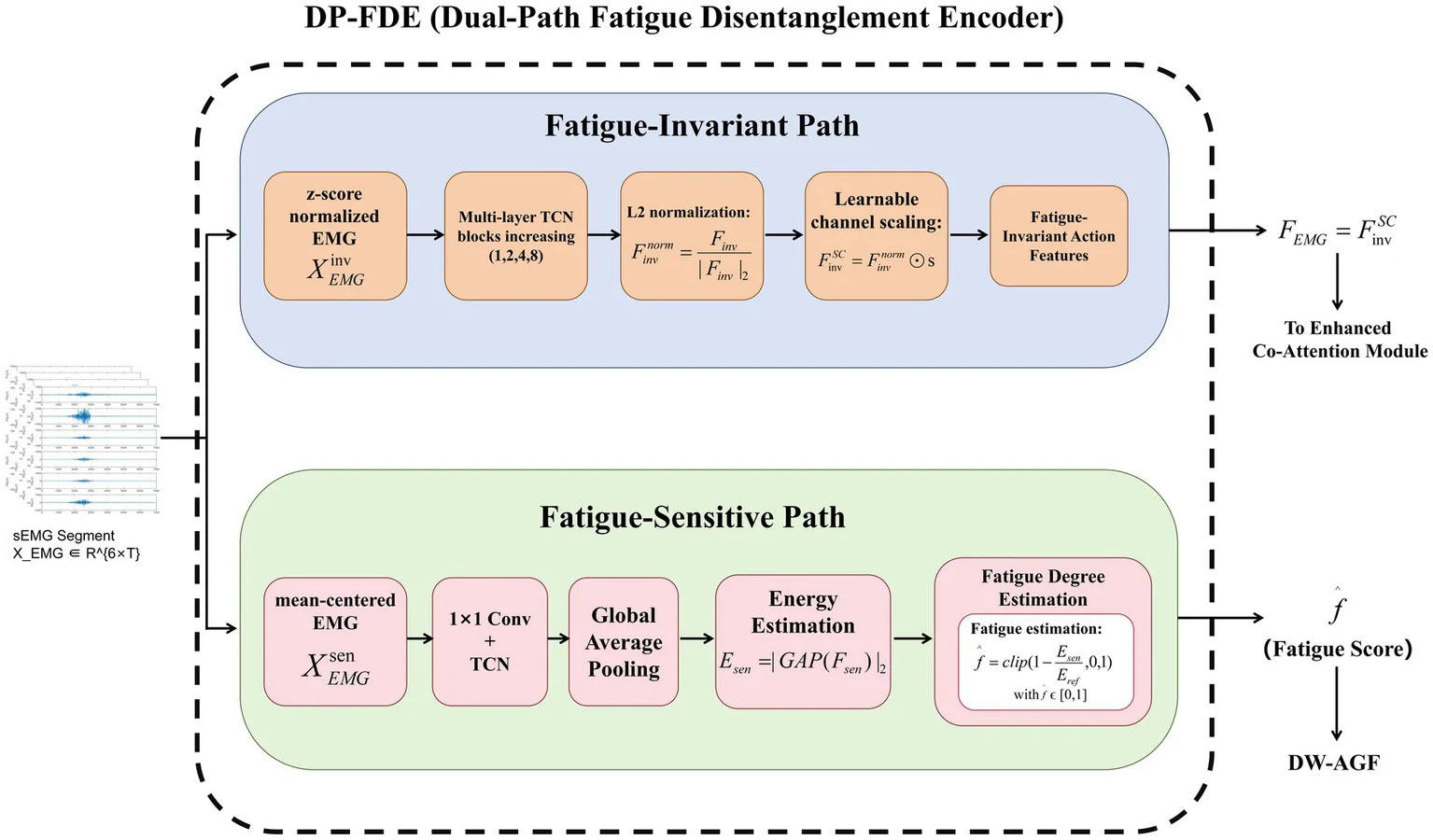
Structure of the DP-FDE module.

To address this issue, a dual-path fatigue disentanglement encoder is proposed, as shown in Figure 2. It contains a fatigue-invariant path and a fatigue-sensitive path. The fatigue-invariant path focuses on extracting action-related features that are relatively stable across fatigue levels. The fatigue-sensitive path preserves amplitude information and estimates fatigue degree from signal energy decay. The outputs of these two paths are used differently: the invariant feature participates in movement classification, while the fatigue estimate is used for adaptive fusion.

#### 2.3.2 Fatigue-invariant path

The fatigue-invariant path receives *z*-score normalized EMG input. For each EMG channel, the signal is normalized by subtracting the mean and dividing by the standard deviation. This operation reduces the influence of global amplitude attenuation caused by fatigue and encourages the network to focus on the temporal pattern and relative activation structure.

The temporal convolution block in the invariant path is defined as follows (Equation 12):


$$H_{l}=\text{GELU}(\mathrm{BN}(\text{Conv}1D_{d_{l}}(H_{l-1})))$$ (12)

where $d_{l}$ is the dilation rate at layer l . A set of increasing dilation rates is used to enlarge the temporal receptive field and model both local muscle activation patterns and longer temporal dependencies. After several temporal convolution blocks, the raw invariant feature $H_{\mathrm{inv}}$ is obtained.

To further reduce the influence of fatigue-induced amplitude scaling, L2 normalization is applied (Equation 13):


$$H_{\mathrm{norm}}=\frac{H_{\mathrm{inv}}}{∥H_{\mathrm{inv}}{∥}_{2}+\varepsilon }$$ (13)

where ε is a small constant used to avoid division by zero. Then, a learnable channel scaling vector β is introduced (Equation 14):


$$Z_{M}=\beta ⊙H_{\mathrm{norm}}$$ (14)

The L2 normalization preserves feature direction while reducing magnitude variation, and the learnable scaling vector allows the model to reweight different muscle channels according to their importance for movement classification. The resulting feature $Z_{M}$ is used as the main EMG action representation.

#### 2.3.3 Fatigue-sensitive path

The fatigue-sensitive path is designed to capture degradation-related information. Unlike the invariant path, it does not perform variance normalization, because fatigue-related amplitude changes should be preserved. The input to this path is mean-centered EMG (Equation 15):


$$X_{M}^{c}=X_{\mathrm{EMG}}-\text{mean}(X_{\mathrm{EMG}})$$ (15)

The fatigue-sensitive feature is extracted by a 1 × 1 convolution followed by temporal convolution (Equation 16):


$$H_{f}=\mathrm{TCN}(\text{Conv}1D_{1\times 1}(X_{M}^{c}))$$ (16)

Then, global average pooling is applied along the temporal dimension, and the sample-level energy statistic is computed (Equation 17):


$$E=∥\mathrm{GAP}(H_{f}){∥}_{2}$$ (17)

A reference energy $E_{\mathrm{ref}}$ is estimated from the running mean energy of the training set. The fatigue degree is then estimated as follows (Equation 18):


$$r_{f}=\text{clip}(1-\frac{E}{E_{\mathrm{ref}}+\varepsilon },,0,,1)$$ (18)

Under the controlled degradation protocol, when the EMG energy statistic is close to the training-derived reference energy, the internal degradation indicator remains low; as imposed amplitude scaling reduces the energy statistic, the indicator increases. This design does not require explicit fatigue labels. It is physiologically motivated by well-documented fatigue-related changes in sEMG amplitude statistics, spectral distribution, median frequency, and muscle-fiber conduction velocity (Cifrek et al., 2009; Lowery et al., 2002; Sun et al., 2022), but the resulting quantity should not be interpreted as a direct physiological fatigue measurement.

In this study, the estimated quantity is used as an internal EMG degradation indicator that guides sample-wise modality reallocation in the fusion module.

#### 2.3.4 Dual-path coordination

The EMG encoder outputs two types of information (Equations 19, 20):


$$Z_{M}=f_{\mathrm{inv}}(X_{\mathrm{EMG}})$$ (19)

$$r_{f}=f_{\text{fatigue}}(X_{\mathrm{EMG}})$$ (20)

Here, $Z_{M}$ is the fatigue-invariant EMG feature used for action classification and cross-modal interaction, whereas $r_{f}$ is the fatigue-sensitive estimate used for adaptive fusion. By separating these two components, DP-FDE reduces the contamination of action features by fatigue-related degradation factors and provides a state signal for the fusion module.

### 2.4 Enhanced co-attention interaction module

Before modality weighting, EEG and EMG features should interact with each other to enhance cross-modal complementarity. EEG and EMG represent different levels of the motor control pathway. EEG contains cortical intention information, while EMG contains peripheral execution information. A co-attention mechanism can help each modality use information from the other modality to refine its representation.

Given the EEG feature $Z_{E}$ and the EMG feature $Z_{M}$ , global statistics are first extracted (Equations 21, 22):


$$g_{E}=[\mathrm{GAP}(Z_{E});\mathrm{GMP}(Z_{E});\mathrm{STD}(Z_{E})]$$ (21)

$$g_{M}=[\mathrm{GAP}(Z_{M});\mathrm{GMP}(Z_{M});\mathrm{STD}(Z_{M})]$$ (22)

where GMP denotes global max pooling and STD denotes standard deviation statistics. These statistics provide compact summaries of the activation distribution of each modality.

Then, cross-modal attention weights are generated (Equations 23, 24):


$$A_{E}=\sigma (\mathrm{LN}({\mathrm{MLP}}_{M\to E}(g_{M})))$$ (23)

$$A_{M}=\sigma (\mathrm{LN}({\mathrm{MLP}}_{E\to M}(g_{E})))$$ (24)

where LN denotes layer normalization. $A_{E}$ is generated from EMG statistics and used to modulate EEG features, while $A_{M}$ is generated from EEG statistics and used to modulate EMG features.

The enhanced features are defined as follows (Equations 25, 26):


$$Z_{E^{\prime }}=Z_{E}+Z_{E}⊙A_{E}$$ (25)

$$Z_{M^{\prime }}=Z_{M}+Z_{M}⊙A_{M}$$ (26)

The residual design preserves the original modality representation while allowing cross-modal enhancement. This prevents excessive suppression of one modality and improves the stability of feature interaction.

### 2.5 Fatigue-aware dynamic weighted adaptive gating fusion

#### 2.5.1 Modality confidence estimation

The reliability of EEG and EMG changes across fatigue states. Therefore, modality confidence is first estimated from the enhanced features. The EEG confidence is calculated as follows (Equation 27):


$$q_{E}=\sigma ({\mathrm{MLP}}_{E}(\mathrm{GAP}(Z_{E^{\prime }})))$$ (27)

The EMG confidence is calculated as follows (Equation 28):


$$q_{M}=\sigma ({\mathrm{MLP}}_{M}(\mathrm{GAP}(Z_{M^{\prime }})))$$ (28)

These confidence scores represent the model’s assessment of each modality’s feature quality. Under normal conditions, EMG usually has high confidence because it directly reflects muscle activation. Under fatigue, EMG confidence decreases as the signal becomes degraded, while EEG confidence remains relatively stable.

#### 2.5.2 Dynamic gating weights

A basic gating weight is generated from the concatenation of EEG and EMG global features (Equation 29):


$$\left[g_{E}^{0},g_{M}^{0}]=\text{Softmax}({\mathrm{MLP}}_{\text{gate}}([\mathrm{GAP}(Z_{E^{\prime }});\mathrm{GAP}(Z_{M^{\prime }})])\right)$$ (29)

The basic weights are then modulated by modality confidence (Equations 30, 31):


$${\tilde{w}}_{E}=\frac{g_{E}^{0}q_{E}}{g_{E}^{0}q_{E}+g_{M}^{0}q_{M}+\varepsilon }$$ (30)

$${\tilde{w}}_{M}=\frac{g_{M}^{0}q_{M}}{g_{E}^{0}q_{E}+g_{M}^{0}q_{M}+\varepsilon }$$ (31)

To explicitly incorporate fatigue information, the fatigue estimate $r_{f}$ from the EMG fatigue-sensitive path is used to increase the EEG weight, with the EMG weight defined as its complement (Equations 32, 33):


$$w_{E}=\text{clip}({\tilde{w}}_{E}+\lambda_{f}r_{f},,w_{\min},,w_{\max})$$ (32)

$$w_{M}=1-w_{E}$$ (33)

Here, $\lambda_{f}$ is the fatigue bias coefficient, and $w_{\min}$ and $w_{\max}$ constrain the range of the EEG weight. This prevents the model from completely ignoring either modality while ensuring that EEG becomes more important when EMG is severely degraded.

#### 2.5.3 Fused feature and classifier

The final fused feature is obtained by weighted summation (Equation 34):


$$Z_{F}=w_{E}Z_{E^{\prime }}+w_{M}Z_{M^{\prime }}$$ (34)

The fused feature is then passed through global average pooling and a fully connected classifier (Equation 35):


$$p=\text{Softmax}(\mathrm{FC}(\mathrm{GAP}(Z_{F})))$$ (35)

The predicted class is the class with the highest probability (Equation 36):


$$\hat{y}=\arg\max_{k}p_{k}$$ (36)

The training objective is the combination of cross-entropy loss and confidence regularization (Equation 37):


$$ℒ={ℒ}_{\mathrm{CE}}+\lambda_{q}{ℒ}_{q}$$ (37)

where ${ℒ}_{\mathrm{CE}}$ is the standard cross-entropy loss and ${ℒ}_{q}$ is a confidence regularization term that encourages the model to maintain reliable modality representations during training. This design allows classification learning and modality quality estimation to be optimized jointly. Unlike additional compensation networks, the proposed design keeps the architecture relatively simple while providing implicit suppression of low-quality modality information.

## 3 Materials

### 3.1 Dataset

Experiments were conducted on the public multimodal dataset released by Jeong et al. (2020), which contains EEG, EMG, and electrooculography signals from 25 healthy right-handed subjects during 11 intuitive upper-extremity movement tasks. The subjects included 15 males and 10 females aged 24–32 years. EEG was recorded from 60 channels, EMG from six muscle channels, and EOG from four channels. Public EEG datasets have been widely used to standardize BCI model evaluation, and the dataset selection and evaluation strategy in this study were also informed by previous public EEG dataset studies (Cho et al., 2017).

This study focuses on fatigue-robust hand grasp recognition. Three representative grasping tasks were selected: spherical grasp., cylindrical grasp., and hook grasp. These movements have clear biomechanical meanings and different muscle activation patterns. For EEG, 19 sensorimotor-related channels were used, including FC1–FC6, Cz, C1–C6, and CP1–CP6. For EMG, six hand- and upper-limb-related muscle channels were used, including extensor carpi ulnaris, extensor digitorum, flexor carpi radialis, flexor carpi ulnaris, biceps brachii, and triceps brachii. EEG and EMG were resampled to 500 Hz after preprocessing, and each movement trial lasted approximately 5 s.

### 3.2 Fatigue simulation

The public dataset does not provide explicit fatigue-level labels. To systematically evaluate the robustness of the proposed model under different fatigue conditions, a synthetic fatigue simulation strategy was adopted. For a given fatigue level l∈[0.1,0.9] , the degraded EMG signal was generated as follows (Equation 38):


$$X_{\mathrm{EMG}}^{\left(l\right)}=(1-l)X_{\mathrm{EMG}}+\eta_{l}$$ (38)

where $\eta_{l}$ is Gaussian noise whose standard deviation is proportional to the signal root mean square value and the fatigue level. The amplitude attenuation term simulates the reduction in muscle activation capacity, while the additive noise term simulates fatigue-related signal quality degradation.

Nine synthetic degradation levels from 10 to 90% were used. Within this controlled perturbation scale, 10% represents a near-normal or mildly degraded condition, whereas 90% represents severe imposed degradation. For brevity, this synthetic degradation level is subsequently referred to throughout the manuscript as the “fatigue level” or “X% fatigue”; this terminology denotes the imposed synthetic EMG degradation condition defined in Equation 38 and does not represent a measured or validated index of physiological muscle fatigue. The simulated level was used only for robustness evaluation and was not used as an explicit class label. Physiological muscle fatigue is a multidimensional and task-dependent process involving both peripheral and central mechanisms; therefore, the present perturbation should not be interpreted as a complete biophysical reproduction of fatigue. Established physiological studies show that fatigue can alter sEMG amplitude behavior and spectral distribution, including spectral compression or shifts toward lower frequencies and reduced muscle-fiber conduction velocity (Cifrek et al., 2009; Lowery et al., 2002; Sun et al., 2022). Accordingly, amplitude attenuation plus additive noise is used here as a controlled surrogate for one practically relevant subset of fatigue-related EMG reliability changes rather than as a universal fatigue biomarker. Similar simulation protocols based on proportional sEMG amplitude attenuation and additive noise have been adopted in previous EEG–sEMG robustness studies, including Gaussian noise-based perturbation and random-noise injection (Yang et al., 2022; Tang et al., 2024; Tang et al., 2025). In particular, Yang et al. (2022) and Tang et al. (2024) reduced sEMG amplitude and added Gaussian noise to construct graded fatigue-related degradation, while Tang et al. (2025) systematically reduced sEMG amplitude and introduced random noise to simulate different fatigue levels. Thus, the current experiments evaluate robustness to selected fatigue-related EMG degradation patterns while retaining a clear boundary from direct physiological-fatigue validation.

### 3.3 Data preprocessing

EEG and EMG signals were preprocessed using standardized procedures. For EEG, a 0.1–50 Hz band-pass filter was applied using a fourth-order Butterworth filter. A 50 Hz notch filter with Q=30 was used to remove power-line interference. Each movement trial from 0 to 5 s was used as one input sample. A baseline window from −0.2 to 0 s before task onset was used for mean correction. Then, *z*-score normalization was applied to each EEG channel.

For EMG, a 20–200 Hz band-pass filter and a 50 Hz notch filter were applied. Different normalization strategies were used for the two EMG paths. The fatigue-invariant path used *z*-score normalization to reduce the effect of amplitude attenuation. The fatigue-sensitive path used only mean centering without variance normalization to preserve amplitude-related fatigue information. These two versions of the same filtered EMG signal were generated during data loading and fed into the corresponding paths of the EMG encoder.

After preprocessing, each sample contained a synchronized EEG segment and EMG segment corresponding to one complete movement trial. The 0–5 s movement interval of each original trial was treated as an indivisible sample, and no overlapping sliding-window segmentation was applied. The EEG and EMG segments belonging to the same movement trial were linked using the same subject–session–trial identifier and were always assigned to the same cross-validation fold.

To prevent information leakage from derived samples, the cross-validation folds were determined using the original trial identifiers before synthetic fatigue generation and data augmentation. All fatigue-degraded EMG versions derived from the same original trial were retained in the fold assigned to that trial. Mixup and time–frequency masking were applied only to the training subset after fold assignment and were not applied to the validation or test subsets (Zhang et al., 2018; Park et al., 2019). Sample-wise baseline correction and normalization were performed independently for each trial, whereas training-dependent quantities, including the reference EMG energy used in the fatigue-sensitive pathway, were estimated exclusively from the training subset and then applied unchanged to the corresponding validation and test subsets.

### 3.4 Comparison methods

To evaluate the effectiveness of FRFNet, seven comparison configurations were considered. E2FNet is an EEG–EMG fusion network using multiscale convolution and cross-modal attention for hand movement intention recognition (Jiang et al., 2024). DCA Fusion is a conventional feature-level fusion method that projects EEG and EMG features into a discriminative correlated space before classification. DMEFNet-adapted is a dense co-attention-based multimodal enhanced fusion network adapted to the present hand-movement task (Shi et al., 2022). A FAConformer-style reimplementation was included as a recent convolutional-Transformer multimodal baseline with frequency-aware attention (He et al., 2024). A decision-based fusion baseline (DBF) represented conventional late fusion of independently decoded EEG and EMG outputs. EEG-only used the MS-ATCN encoder without EMG input, whereas EMG-only used the EMG encoder without EEG input or adaptive fusion.

All comparison methods were reimplemented under the same settings. Experiments were conducted in PyCharm using Python 3.9 and PyTorch. A within-subject nested stratified five-fold cross-validation strategy was adopted independently for each participant. The original movement trial, rather than an augmented sample or a temporal subwindow, was used as the basic partitioning unit. In each outer iteration, four folds were used as the development set and the remaining fold was held out as the independent test set. The synchronized EEG and EMG signals from the same trial were treated as one paired multimodal sample and were never assigned to different folds.

The inner cross-validation procedure was performed exclusively within the outer development set and was used for model selection, early stopping, and validation. The outer test fold remained completely isolated during training and hyperparameter selection. Synthetic fatigue generation and training-time augmentation were performed only after the original trials had been assigned to their respective folds. Therefore, neither augmented versions nor fatigue-degraded versions derived from an outer-test trial were included in the training or validation data. The reference energy of the fatigue-sensitive EMG pathway and all other training-dependent quantities were estimated from the corresponding training subset only.

The stratification was performed at the trial level rather than the recording-session level. Thus, although no identical trial or temporally overlapping segment was shared between the training and test sets, different trials recorded in the same session could occur in both sets. Accordingly, the current protocol evaluates within-subject trial-level generalization and should not be interpreted as session-independent evaluation.

In addition to the within-subject protocol, strict leave-one-subject-out (LOSO) evaluation was conducted to assess unseen-subject generalization. In each of the 25 LOSO folds, all trials from one participant were reserved for testing and the remaining 24 participants were used for model development. Data from the held-out participant were excluded from training, validation, normalization-statistic estimation, reference-energy estimation, augmentation, and hyperparameter selection. The same subject partition was used for all methods and fatigue levels. The strict LOSO analysis was conducted as a separate cross-subject experiment and was not nested within or derived from the within-subject five-fold partitions. Accordingly, the within-subject protocol evaluates trial-level generalization within each participant, whereas LOSO evaluates unseen-subject generalization; neither protocol should be interpreted as session-independent evaluation unless recording sessions themselves are held out.

AdamW was used as the optimizer, with a batch size of 32 and a maximum of 70 epochs. A cosine annealing learning rate scheduler and early stopping based on validation macro-F1 were applied. All results were calculated independently for 25 subjects and reported as mean ± standard deviation.

The default hyperparameters were fixed across all participants and fatigue levels. In MS-ATCN, the short-, medium-, and long-range branches used dilation schedules of {1, 2, 4}, {2, 4, 8}, and {4, 8, 16}, respectively. The fatigue-bias coefficient *λ_f_* was 0.5, the confidence-regularization weight *λ_q_* was 0.01, the confidence-estimation hidden dimension was 64, and the temporal kernel size of the fusion decoder was 5. Their sensitivity was evaluated through one-factor-at-a-time perturbation while all remaining settings were fixed. The outer test folds were not used for hyperparameter selection.

### 3.5 Evaluation metrics

Classification accuracy was used as the main evaluation metric. For the s -th subject, accuracy was defined as follows (Equation 39):


$${\mathrm{Acc}}_{s}=\frac{N_{\text{correct}}}{N_{\text{total}}}$$ (39)

where $N_{\text{correct}}$ is the number of correctly classified samples and $N_{\text{total}}$ is the total number of test samples.

The average accuracy across all subjects was calculated as follows (Equation 40):


$$\bar{\mathrm{Acc}}=\frac{1}{S}\sum_{s=1}^{S}{\mathrm{Acc}}_{s}$$ (40)

The standard deviation was calculated as follows (Equation 41):


$$\mathrm{SD}=\sqrt{\frac{1}{S-1}\sum_{s=1}^{S}{\left({\mathrm{Acc}}_{s}-\bar{\mathrm{Acc}}\right)}^{2}}$$ (41)

where S=25 is the number of subjects. Therefore, all experimental results are reported in the form of mean ± standard deviation.

To provide a broader multiclass evaluation, macro-precision, macro-recall, macro-F1, and Cohen’s *κ* were also reported. For *K* classes, macro-precision and macro-recall are the unweighted means of the class-specific precision and recall values, and macro-F1 is the unweighted mean of the class-specific F1 scores. Cohen’s *κ* was calculated as *κ* = (*p*_*o* − *p*_*e*)/(1 − *p*_*e*), where *p*_*o* is the observed agreement and *p*_*e* is the expected agreement by chance. Because the three grasp classes were balanced, macro-recall equals overall accuracy. A compact summary of these metrics is provided in Table 1.

**Table 1:** Compact summary of additional multiclass metrics across the nine fatigue levels.

Method	Mean precision (%)	Mean recall (%)	Mean F1 (%)	Mean *κ*	F1 at 90% (%)	*κ* at 90%
Within-subject nested stratified five-fold cross-validation						
**FRFNet**	**75.7**	**74.0**	**73.0**	**0.615**	**57.5**	**0.398**
E2FNet	68.4	65.8	63.7	0.484	42.3	0.212
DCA Fusion	65.5	62.7	60.2	0.439	39.7	0.171
DMEFNet-adapted	67.5	64.7	62.6	0.472	43.2	0.226
FAConformer-style	62.6	58.5	55.4	0.379	42.9	0.229
DBF	52.0	49.7	45.3	0.246	34.0	0.112
Strict leave-one-subject-out (LOSO)						
**FRFNet**	**67.9**	**65.0**	**63.2**	**0.476**	**54.1**	**0.357**
E2FNet	46.7	44.1	38.8	0.158	27.6	0.019
DCA Fusion	56.5	52.6	49.0	0.290	38.1	0.143
DMEFNet-adapted	58.6	53.9	51.3	0.310	39.7	0.165
FAConformer-style	60.7	56.3	54.0	0.343	50.0	0.298
DBF	59.6	54.1	51.9	0.314	47.5	0.254

Mean values are arithmetic averages across the nine fatigue levels. The three classes were balanced; therefore, macro-recall equals overall accuracy. The reported macro-precision, macro-recall, macro-F1, and Cohen’s *κ* values are summarized consistently for both evaluation protocols.

### 3.6 Statistical analysis

Statistical analyzes were conducted using the subject-level classification accuracies of the 25 participants. For each participant, recognition method, and fatigue level, the reported subject-level accuracy was used as one statistical observation. Individual cross-validation folds were not treated as independent observations, thereby avoiding pseudo-replication.

A two-way repeated-measures analysis of variance was conducted separately for the within-subject and strict LOSO protocols, with recognition method and fatigue level as within-participant factors. The method factor included FRFNet and five comparison models, while the fatigue factor included nine levels from 10 to 90%. Greenhouse–Geisser-adjusted degrees of freedom and partial *η*^2^ were reported for the repeated-measures effects.

Following the omnibus analysis, two-sided paired *t*-tests compared FRFNet with each baseline at every fatigue level. Holm correction was applied across 45 planned comparisons within each protocol, and the standardized mean difference *d*_*av* was reported as the effect size. For concise significance reporting, Holm-adjusted *p* values were grouped into the following categories: *p* > 0.05, not significant; *p* < 0.05, significant; *p* < 0.01, stronger statistical evidence; *p* < 0.005, still stronger statistical evidence; and *p* < 0.001, the strongest category used in this study. Thus, smaller *p*-value thresholds indicate progressively stronger evidence against the null hypothesis.

### 3.7 Computational complexity and inference-efficiency evaluation

Computational efficiency was evaluated for FRFNet, E2FNet, DMEFNet-adapted, FAConformer-style, and DBF under a unified deployment benchmark. The tests were conducted using Python 3.9.23 and PyTorch 2.7.1 with CUDA 12.6 on an NVIDIA GeForce RTX 4060 Laptop GPU. All models received FP32 EEG and EMG tensors with dimensions of [B, 19, 2,500] and [B, 6, 2,500], respectively. Trainable parameter count, FLOPs, serialized model size, single-trial latency, 95th-percentile latency, and peak GPU memory were recorded.

Single-trial latency was measured with a batch size of one after 30 warm-up passes using 200 repeated forward passes. The reported timing covers network inference only and excludes signal acquisition, filtering, disk I/O, metric calculation, communication, and actuator response. FLOPs were recomputed from the exact model implementations over the complete deployment inference path using the same input dimensions for all neural models. DCA Fusion was not assigned neural-network parameter or FLOP values because it is a handcrafted-feature and linear-classification pipeline.

### 3.8 Channel-wise occlusion analysis

To quantify the predictive contribution of individual EEG electrodes and EMG channels, a channel-wise occlusion analysis was performed using the held-out test data from the within-subject nested cross-validation protocol. The trained model parameters remained fixed during the analysis. For each EEG channel independently, the complete temporal sequence was set to zero after channel-wise *z*-score standardization, while all remaining EEG channels were kept unchanged. For each EMG channel, the corresponding temporal sequence was simultaneously set to zero in both branch-specific inputs: the *z*-score-normalized input of the fatigue-invariant pathway and the mean-centered input of the fatigue-sensitive pathway. Because both branch-specific inputs were centered before occlusion, zero represented the channel-wise mean level rather than an arbitrary extreme value. The trained model parameters, preprocessing procedure, normalization settings, and training-derived reference EMG energy remained fixed throughout the occlusion analysis.


$$\mathrm{\Delta Ac}c_{c}=\mathrm{Ac}c_{\text{full}}-\mathrm{Ac}c_{\text{occluded},c}$$

where the full-input accuracy denotes the performance obtained using all channels and the occluded-channel accuracy denotes the performance after independently occluding channel c. A larger positive accuracy reduction indicates a greater predictive contribution of the corresponding channel. The analysis was conducted separately at 10, 50, and 90% synthetic fatigue. Subject-level values were averaged across the 25 participants and are reported as mean ± standard deviation.

### 3.9 Comparison with alternative fusion strategies

To isolate the contribution of the proposed fatigue-aware fusion mechanism, three representative fusion strategies were evaluated using identical EEG and EMG encoders, enhanced co-attention modules, classifiers, optimization settings, data partitions, and random seeds. The AdamW optimizer, a batch size of 32, and 70 training epochs were used for all configurations. Only the multimodal fusion operation was changed.

Feature Concatenation directly concatenated the encoded EEG and EMG representations, followed by a fully connected classifier, without explicit modality weighting. Feature-Dynamic Gating generated sample-dependent modality weights from the globally pooled EEG and EMG representations using an MLP and softmax operation, but did not incorporate modality-confidence scores or the fatigue estimate. The complete DW-AGF jointly incorporated feature-dependent gating, modality confidence, and the explicit fatigue-bias term generated from the DP-FDE fatigue estimate.

All three configurations were evaluated across the complete 10–90% synthetic fatigue range. The held-out outer test folds were not used for hyperparameter selection, model development, or early stopping.

## 4 Results

### 4.1 Recognition performance under different fatigue levels

Table 2 summarizes classification accuracy under the within-subject and strict LOSO protocols. Overall, FRFNet achieved the highest accuracy across the evaluated fatigue levels and showed a slower performance decline than the comparison methods. Under 10 and 20% fatigue in the within-subject protocol, FRFNet obtained accuracies of 89.9 ± 3.1% and 88.7 ± 3.4%, respectively, indicating that the model can effectively exploit reliable EMG execution information when signal degradation is limited.

**Table 2:** Classification accuracy (%) of the evaluated models under 10–90% synthetic fatigue using within-subject and strict LOSO protocols.

Method	10%	20%	30%	40%	50%	60%	70%	80%	90%
Within-subject nested stratified five-fold cross-validation									
**FRFNet**	**89.9 ± 3.1**	**88.7 ± 3.4**	**82.3 ± 4.0**	**78.1 ± 4.5**	**73.0 ± 4.8**	**67.8 ± 5.5**	**64.3 ± 6.1**	**62.1 ± 6.8**	**60.0 ± 7.6**
E2FNet	87.2 ± 3.5	85.8 ± 3.8	76.8 ± 4.6	70.6 ± 5.0	63.5 ± 5.3	57.5 ± 6.0	53.2 ± 6.8	50.4 ± 7.5	47.3 ± 8.1
DCA Fusion	84.9 ± 3.8	82.0 ± 4.1	74.2 ± 4.8	67.4 ± 5.2	60.0 ± 5.7	53.7 ± 6.4	50.5 ± 7.2	47.0 ± 8.1	44.8 ± 9.4
DMEFNet-adapted	83.2 ± 3.9	80.4 ± 4.0	75.2 ± 4.6	69.3 ± 5.1	63.5 ± 5.4	57.1 ± 6.2	54.0 ± 7.0	51.2 ± 7.8	48.3 ± 9.0
FAConformer-style	77.2 ± 8.1	71.8 ± 8.4	63.9 ± 7.9	58.6 ± 7.7	54.6 ± 7.8	52.0 ± 8.3	50.8 ± 8.0	49.5 ± 8.2	48.5 ± 7.9
DBF	62.4 ± 9.3	59.3 ± 9.8	54.6 ± 9.9	50.6 ± 9.1	48.2 ± 8.4	45.6 ± 7.1	43.7 ± 6.5	41.7 ± 6.6	40.8 ± 6.4
EEG-only	70.2 ± 4.2	70.2 ± 4.2	70.2 ± 4.2	70.2 ± 4.2	70.2 ± 4.2	70.2 ± 4.2	70.2 ± 4.2	70.2 ± 4.2	70.2 ± 4.2
EMG-only	89.3 ± 3.9	87.7 ± 4.4	80.4 ± 4.8	68.9 ± 5.7	57.0 ± 6.6	48.5 ± 7.3	44.0 ± 8.1	41.3 ± 8.8	39.8 ± 9.8
Strict leave-one-subject-out (LOSO)									
**FRFNet**	**71.6 ± 10.3**	**71.1 ± 10.0**	**70.0 ± 10.0**	**68.1 ± 10.6**	**65.8 ± 10.4**	**62.7 ± 9.6**	**60.2 ± 9.7**	**58.1 ± 10.5**	**57.0 ± 10.1**
E2FNet	61.1 ± 10.7	56.4 ± 11.6	50.6 ± 12.0	45.1 ± 9.7	40.6 ± 6.6	37.5 ± 4.3	35.7 ± 2.9	35.0 ± 2.6	34.8 ± 3.5
DCA Fusion	61.0 ± 11.5	60.5 ± 11.2	59.0 ± 10.8	56.5 ± 10.5	53.2 ± 9.6	49.8 ± 9.0	46.5 ± 8.5	44.0 ± 8.0	42.8 ± 7.8
DMEFNet-adapted	62.4 ± 12.8	61.6 ± 12.8	60.2 ± 12.1	58.2 ± 10.7	54.7 ± 9.8	50.9 ± 9.2	47.4 ± 8.8	45.3 ± 7.9	44.2 ± 7.6
FAConformer-style	59.7 ± 9.8	59.3 ± 9.8	58.5 ± 10.2	57.2 ± 10.1	56.3 ± 9.5	54.8 ± 9.4	53.9 ± 9.1	53.6 ± 9.3	53.1 ± 8.8
DBF	60.1 ± 9.6	58.4 ± 9.1	56.2 ± 8.5	54.8 ± 7.8	53.7 ± 7.3	52.0 ± 7.6	51.1 ± 8.0	50.6 ± 8.5	50.3 ± 8.2

Values are mean ± sample standard deviation across 25 participants. Bold values indicate the proposed FRFNet model, provided as the reference for comparison with each baseline method. EEG-only and EMG-only were evaluated only under the within-subject protocol.

**Figure 3:**
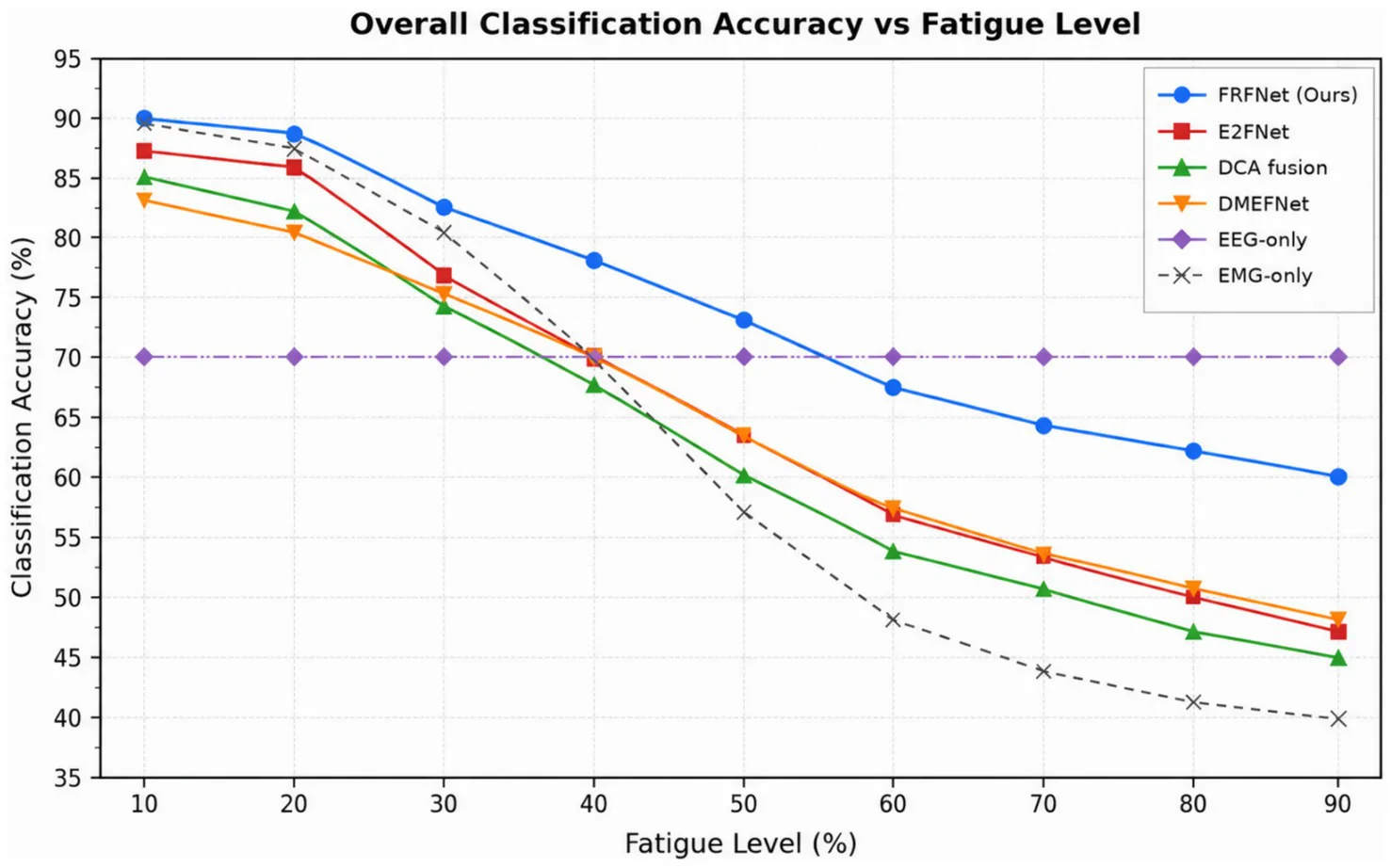
Classification accuracy under different fatigue levels.

As fatigue increased, the advantage of FRFNet became more evident. At 30, 40, and 50% fatigue, FRFNet achieved 82.3 ± 4.0%, 78.1 ± 4.5%, and 73.0 ± 4.8%, respectively. Under 90% severe fatigue, FRFNet still achieved 60.0 ± 7.6%. EEG-only remained stable because synthetic fatigue was applied only to EMG, whereas EMG-only dropped from 89.3 ± 3.9% at 10% fatigue to 39.8 ± 9.8% at 90% fatigue. Figure 3 further shows that FRFNet maintains a more gradual decline than the comparison methods. Subject-level statistical analyzes are reported in Section 4.2 and Table 3.

**Table 3:** Subject-level statistical analyzes under the within-subject and strict LOSO protocols.

Protocol	Effect/comparison	Summary statistic	*p* significance category	Effect size	Significant levels	GG ε
Panel A. Six-method two-way repeated-measures ANOVA						
Within-subject	Method	*F* = 108.77 (2.64, 63.35)	*p* < 0.001	partial *η*^2^ = 0.819	–	0.528
	Fatigue level	*F* = 711.67 (1.52, 36.42)	*p* < 0.001	partial *η*^2^ = 0.967	–	0.190
	Method × fatigue	*F* = 35.71 (4.53, 108.73)	*p* < 0.001	partial *η*^2^ = 0.598	–	0.113
Strict LOSO	Method	*F* = 65.12 (3.81, 91.44)	*p* < 0.001	partial *η*^2^ = 0.731	–	0.761
	Fatigue level	*F* = 118.45 (1.46, 35.04)	*p* < 0.001	partial *η*^2^ = 0.831	–	0.183
	Method × fatigue	*F* = 18.97 (5.88, 141.12)	*p* < 0.001	partial *η*^2^ = 0.441	–	0.147
Panel B. Holm-adjusted paired comparisons: FRFNet versus each baseline						
Within-subject	E2FNet	Δ*Acc* = +2.7/+9.5/+12.7	*p* < 0.005	*d*_*av* = 0.80–1.88	9/9	–
	DCA Fusion	Δ*Acc* = +5.0/+13.0/+15.2	*p* < 0.001	*d*_*av* = 1.44–2.47	9/9	–
	DMEFNet-adapted	Δ*Acc* = +6.7/+9.5/+11.7	*p* < 0.001	*d*_*av* = 1.40–2.24	9/9	–
	FAConformer-style	Δ*Acc* = +12.7/+18.4/+11.5	*p* < 0.001	*d*_*av* = 1.48–3.11	9/9	–
	DBF	Δ*Acc* = +27.5/+24.8/+19.2	*p* < 0.001	*d*_*av* = 2.73–4.01	9/9	–
Strict LOSO	E2FNet	Δ*Acc* = +10.5/+25.1/+22.2	*p* < 0.001	*d*_*av* = 1.00–3.40	9/9	–
	DCA Fusion	Δ*Acc* = +10.6/+12.6/+14.2	*p* < 0.01	*d*_*av* = 0.97–1.57	9/9	–
	DMEFNet-adapted	Δ*Acc* = +9.2/+11.0/+12.8	*p* < 0.001	*d*_*av* = 0.79–1.43	9/9	–
	FAConformer-style	Δ*Acc* = +11.9/+9.5/+3.9	*p* < 0.05	*d*_*av* = 0.41–1.19	9/9	–
	DBF	Δ*Acc* = +11.5/+12.1/+6.7	*p* < 0.05	*d*_*av* = 0.72–1.49	9/9	–

Δ*Acc* values are FRFNet minus baseline accuracy at 10, 50, and 90% fatigue. Holm correction was applied across 45 paired *t*-test comparisons within each protocol. Significance categories were reported as *p* > 0.05 (not significant), *p* < 0.05, *p* < 0.01, *p* < 0.005, and *p* < 0.001, with smaller thresholds indicating progressively stronger statistical evidence. For Panel B, the listed category is the least stringent threshold satisfied by all nine Holm-adjusted comparisons for that baseline. Significant levels denotes the number of fatigue levels with Holm-adjusted *p* < 0.05.

### 4.2 Statistical significance analysis

These results demonstrate that the advantage of FRFNet is supported at the participant level and that the evaluated methods exhibit significantly different degradation trajectories as fatigue increases.

Under both the within-subject and strict LOSO protocols, Holm-adjusted paired *t*-tests favored FRFNet over all five baselines at all nine fatigue levels. The corresponding effect-size ranges and Holm-adjusted significance categories are summarized in Table 3.

The strict LOSO analysis also showed significant effects of method, *F*(3.81, 91.44) = 65.12, *p* < 0.001, partial *η*^2^ = 0.731; fatigue level, *F*(1.46, 35.04) = 118.45, *p* < 0.001, partial *η*^2^ = 0.831; and the method-by-fatigue interaction, *F*(5.88, 141.12) = 18.97, *p* < 0.001, partial *η*^2^ = 0.441.

Separate six-method repeated-measures ANOVA models were conducted for the within-subject and strict LOSO protocols. Under the within-subject protocol, significant effects were observed for method, *F*(2.64, 63.35) = 108.77, *p* < 0.001, partial *η*^2^ = 0.819; fatigue level, *F*(1.52, 36.42) = 711.67, *p* < 0.001, partial *η*^2^ = 0.967; and the method-by-fatigue interaction, *F*(4.53, 108.73) = 35.71, *p* < 0.001, partial *η*^2^ = 0.598.

### 4.3 Performance degradation trend

To further analyze robustness to synthetic fatigue-related EMG degradation, the reduction in mean accuracy from 10 to 90% synthetic fatigue was compared across the four EEG–EMG fusion methods. FRFNet decreased by 29.9 percentage points, from 89.9 to 60.0%. In comparison, E2FNet decreased by 39.9 percentage points, DCA Fusion by 40.1 percentage points, and DMEFNet by 34.9 percentage points. The significant method-by-fatigue interaction further confirmed that these degradation trajectories differed statistically. These results indicate that fatigue-aware adaptive fusion effectively reduces the rate of performance degradation caused by progressively deteriorated (synthetically degraded) EMG signals.

### 4.4 Dynamic gating weights

To verify whether DW-AGF performs fatigue-aware modality adjustment, the average EEG and EMG fusion weights were analyzed across synthetic fatigue levels. At 10% fatigue, the EMG weight was 0.519, higher than the EEG weight of 0.481, indicating that the model relied more on EMG when the signal quality was high. As fatigue increased, the EMG weight gradually decreased, while the EEG weight increased. The two weights crossed at approximately 40% fatigue, showing a transition of modality dominance. From 50 to 70% fatigue, EEG became the dominant modality, with its weight reaching about 0.527.

**Figure 4:**
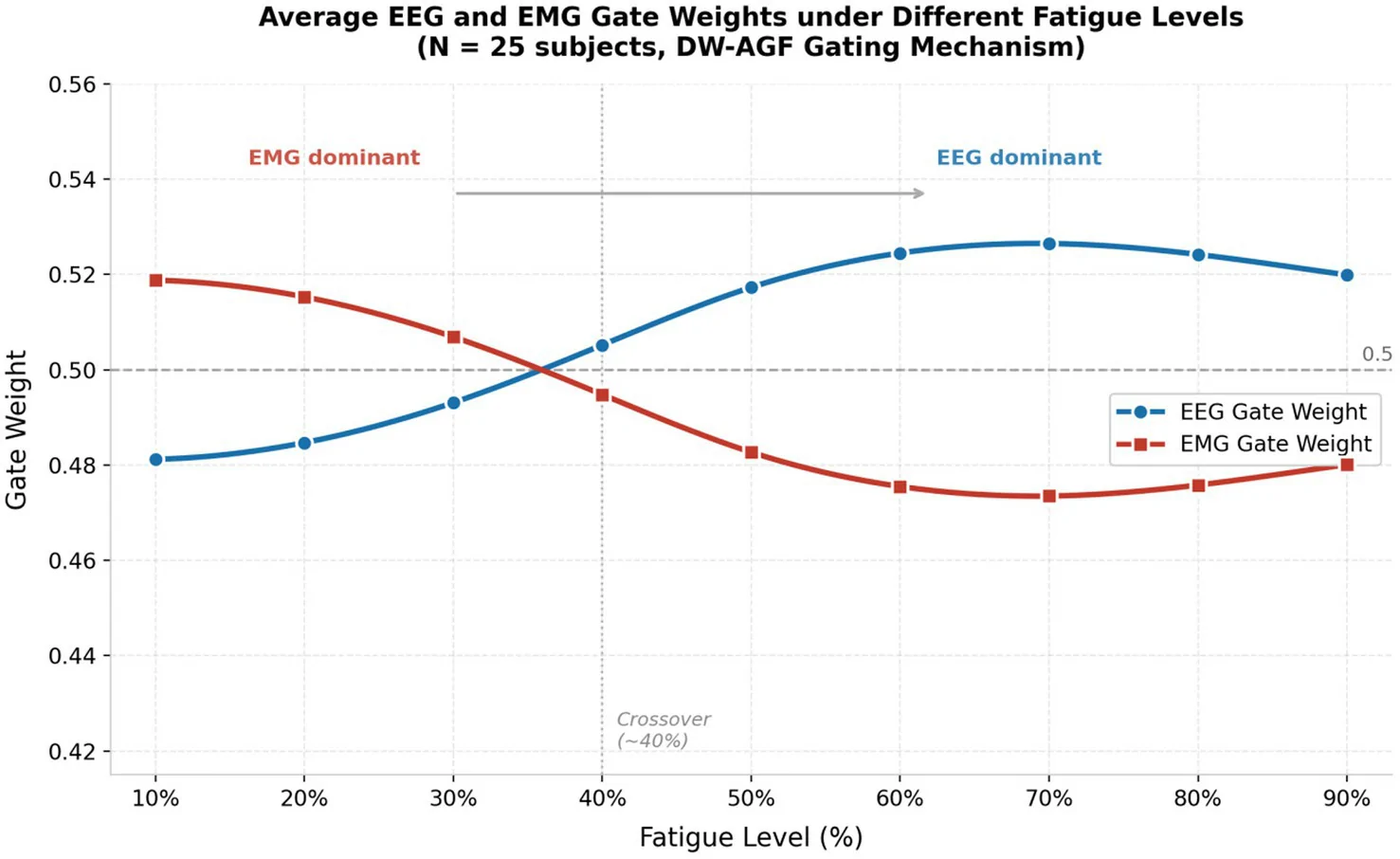
Dynamic gating weights under different fatigue levels.

At 70–90% fatigue, the EEG weight slightly decreased and the EMG weight slightly increased. This may be caused by extreme signal degradation, where additive noise becomes comparable to the attenuated EMG signal and changes the feature energy perceived by the gating network. Nevertheless, EEG remained dominant overall. As shown in Figure 4, DW-AGF does not use fixed modality weights; instead, it adaptively shifts from EMG-dominant fusion under low fatigue to EEG-dominant fusion under severe fatigue. This trend is consistent with the physiological roles of EEG and EMG and supports the effectiveness of adaptive weighting in multimodal fusion.

### 4.5 Modality confidence analysis

**Figure 5:**
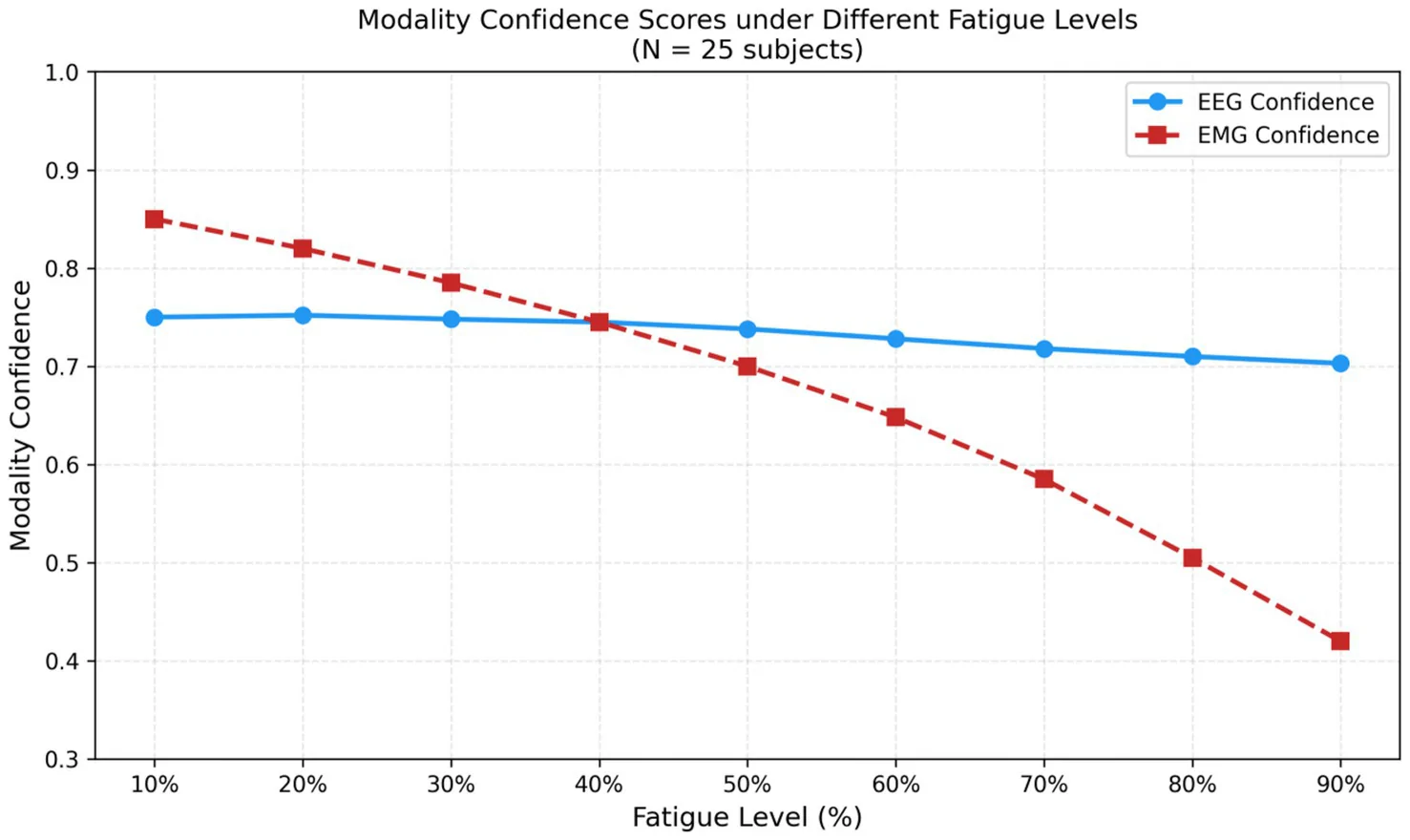
Modality confidence scores under controlled synthetic EMG degradation.

The modality confidence scores were also analyzed, as shown in Figure 5. EEG confidence remained relatively stable across degradation levels, ranging approximately from 0.70 to 0.75. In contrast, EMG confidence progressively decreased as the imposed EMG degradation increased, from about 0.85 under low degradation to about 0.42 under 90% degradation. The direction of this response is consistent with the controlled perturbation and is physiologically plausible because muscle fatigue can alter sEMG amplitude statistics, spectral content, muscle-fiber conduction velocity, and overall signal reliability (Cifrek et al., 2009; Lowery et al., 2002; Sun et al., 2022). However, because the present dataset provides no independent physiological fatigue reference, the learned confidence score is interpreted as a degradation-consistency measure rather than a validated physiological fatigue score.

The parallel decline in EMG confidence and its fusion weight supports the internal consistency of DW-AGF with the imposed signal-quality deterioration. When EMG quality is high, it contributes more to the fused representation; when the controlled degradation becomes stronger, its confidence and fusion contribution are reduced. This behavior helps prevent low-quality EMG features from dominating the classification decision, but it does not establish correlation with genuine physiological fatigue and should not be interpreted as a clinical fatigue scale.

### 4.6 Ablation study

To evaluate the contribution of each core module, three ablation experiments were conducted. In each experiment, one module was removed while all other settings remained unchanged. The tested variants included FRFNet without MS-ATCN, FRFNet without DP-FDE, and FRFNet without DW-AGF.

**Figure 6:**
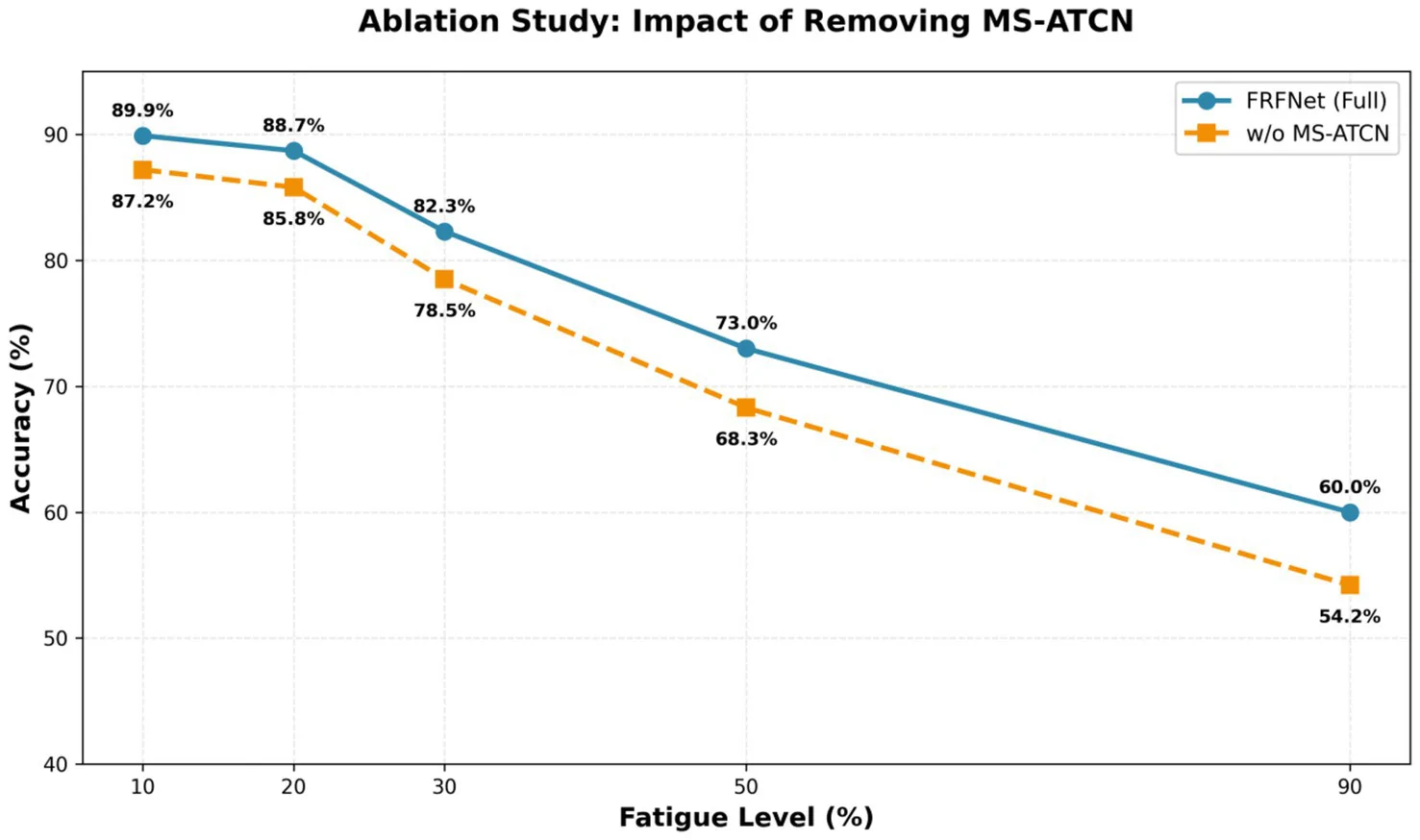
Ablation result of MS-ATCN.

Removing MS-ATCN reduced the mean accuracy across the five representative synthetic degradation levels of 10, 20, 30, 50, and 90% from 78.8 to 74.8%, corresponding to a decrease of 4.0 percentage points. This result is shown in Figure 6. The accuracy decreased from 89.9 to 87.2% at 10% degradation, from 73.0 to 68.3% at 50% degradation, and from 60.0 to 54.2% at 90% degradation. These results indicate that multiscale EEG temporal modeling contributes to stable recognition, with its contribution becoming more pronounced as EMG degradation increases.

**Figure 7:**
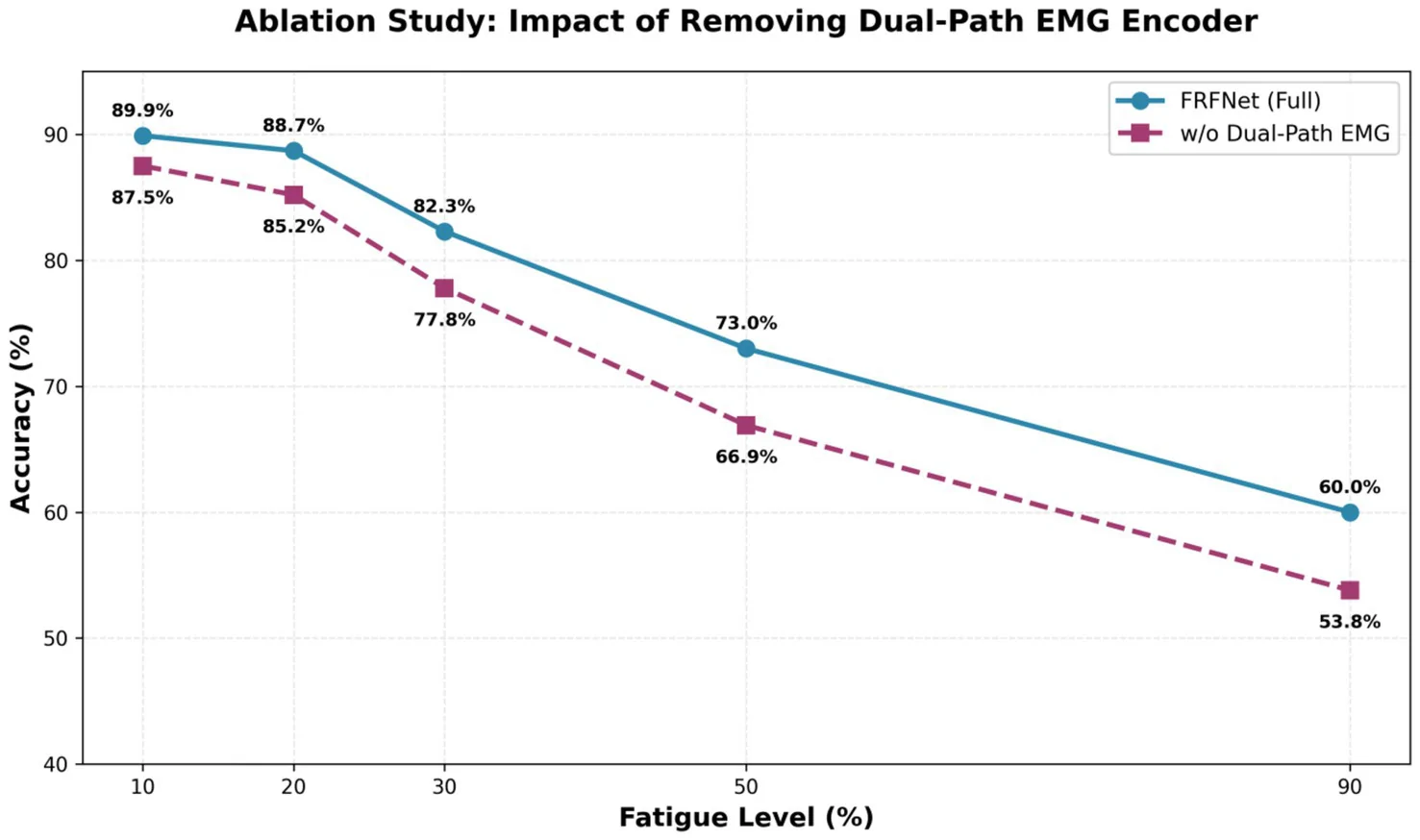
Ablation result of DP-FDE.

Removing DP-FDE reduced the mean accuracy across the five representative degradation levels to 74.2%, corresponding to a decrease of 4.5 percentage points relative to the full FRFNet. As shown in Figure 7, the performance reduction became more evident under severe degradation. At 90% degradation, the accuracy decreased from 60.0 to 53.8%, corresponding to a reduction of 6.2 percentage points. This result demonstrates that separating relatively degradation-invariant action information from degradation-sensitive EMG information improves the stability of EMG representation and provides useful degradation guidance for DW-AGF.

**Figure 8:**
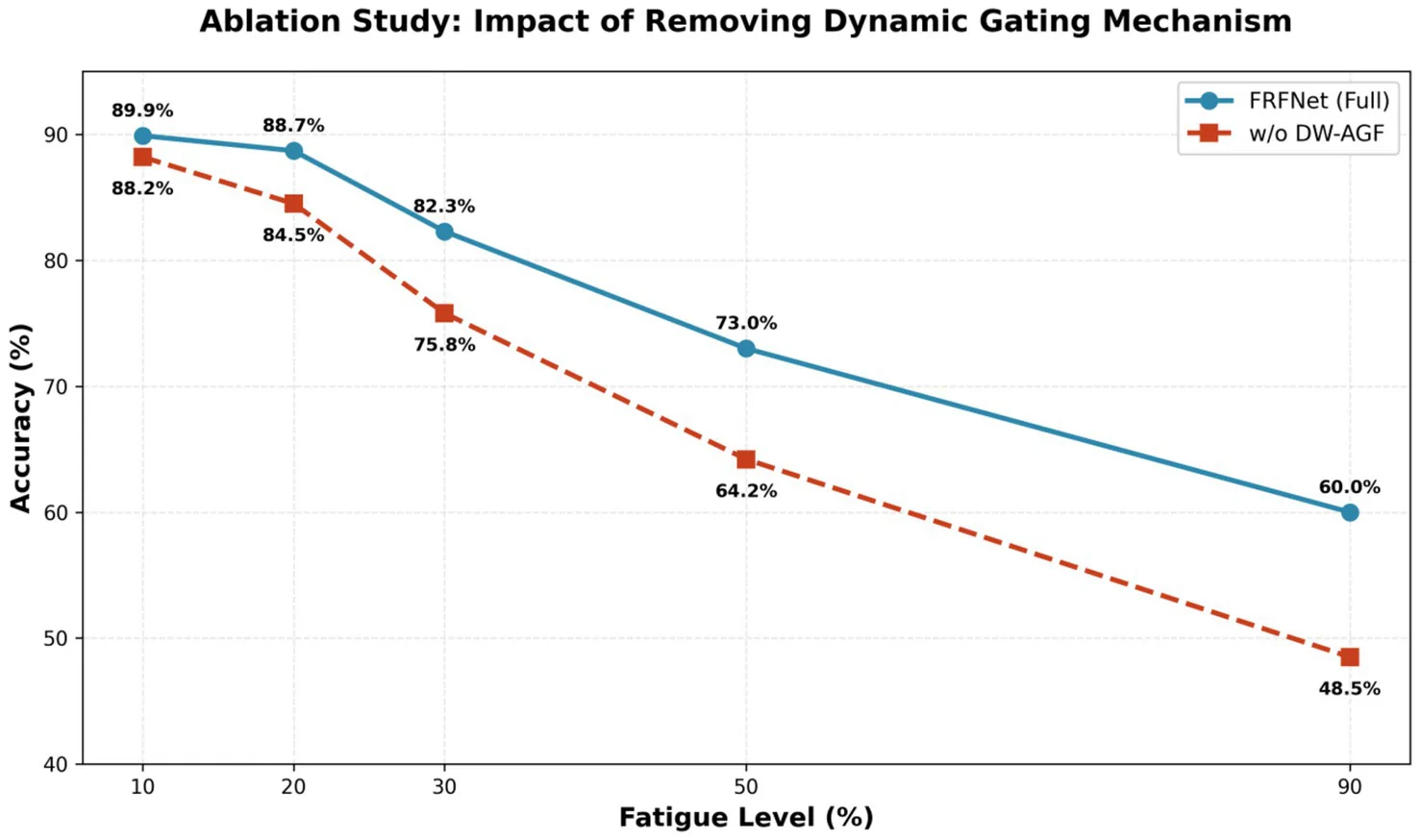
Ablation result of DW-AGF.

Removing DW-AGF caused the largest performance decrease. The mean accuracy across the five representative degradation levels decreased from 78.8 to 72.2%, corresponding to a reduction of 6.5 percentage points. As shown in Figure 8, at 90% severe degradation, the accuracy decreased from 60.0 to 48.5%, corresponding to a reduction of 11.5 percentage points. These results indicate that fatigue-aware dynamic gating is the most influential core component under severe EMG degradation. Without DW-AGF, the model cannot sufficiently adjust the relative contributions of EEG and EMG according to modality reliability.

Overall, all three core modules contributed to the final performance. MS-ATCN improved multiscale EEG representation, DP-FDE improved degradation-aware EMG modeling, and DW-AGF provided reliability-aware cross-modal weighting. Among these modules, DW-AGF produced the largest performance reduction when removed, particularly under severe synthetic degradation.

### 4.7 Cross-subject generalization and extended evaluation

**Figure 9:**
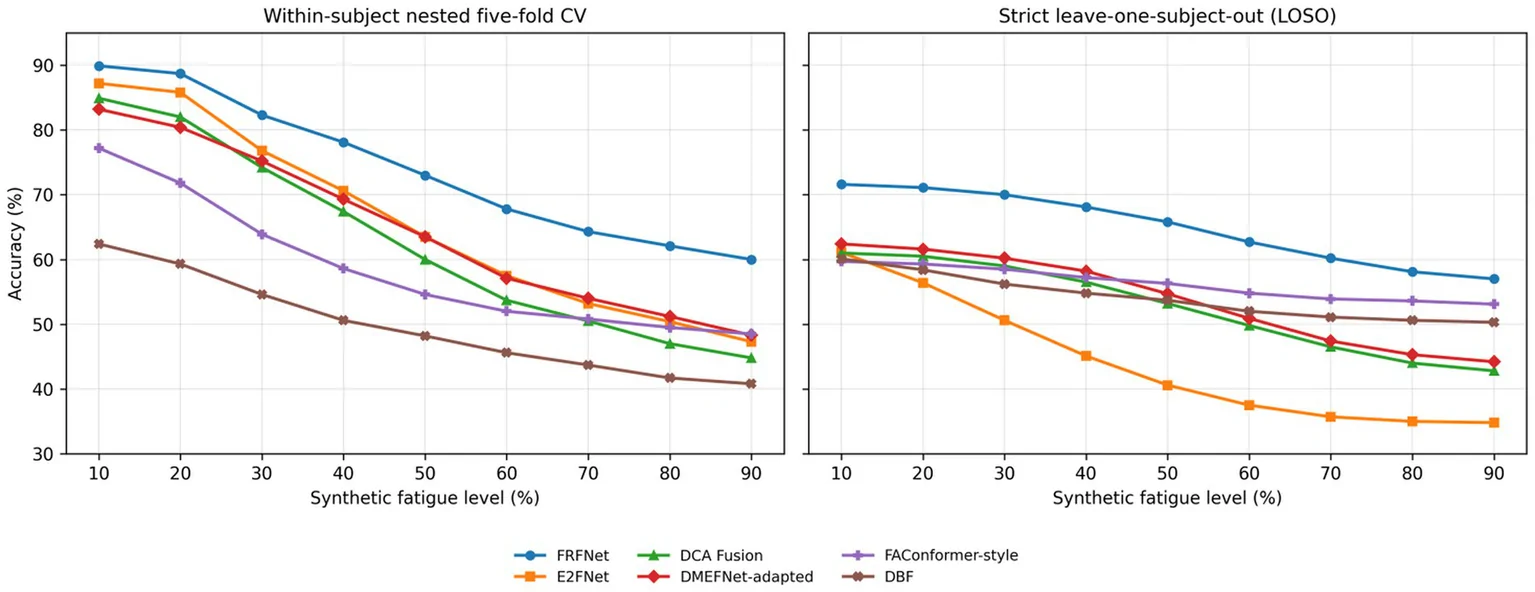
Accuracy of six methods across synthetic fatigue levels under **(a)** within-subject nested five-fold cross-validation and **(b)** strict LOSO evaluation.

Strict LOSO evaluation confirmed that the advantage of FRFNet was not restricted to subject-specific calibration. As summarized in Table 2 and Figure 9, FRFNet achieved 71.6 ± 10.3%, 65.8 ± 10.4%, and 57.0 ± 10.1% accuracy at 10, 50, and 90% synthetic fatigue, respectively. At 90% fatigue, the closest competing method was FAConformer-style at 53.1 ± 8.8%, followed by DBF at 50.3 ± 8.2%, DMEFNet-adapted at 44.2 ± 7.6%, DCA Fusion at 42.8 ± 7.8%, and E2FNet at 34.8 ± 3.5%.

Under the strict LOSO protocol, FRFNet achieved the highest mean accuracy among the evaluated methods. Although its LOSO performance was lower than its within-subject performance, the LOSO results demonstrate that FRFNet retained substantial discriminative capability when tested on completely unseen participants. Because the within-subject and LOSO protocols differ in both subject composition and effective training-set size, they were treated as separate evaluation settings rather than directly compared using the simple difference between their mean accuracies. The additional metrics in Table 1 showed the same overall pattern. At 90% fatigue under LOSO, FRFNet achieved a macro-F1 of 54.1% and *κ* = 0.357, compared with 50.0% and *κ* = 0.298 for FAConformer-style.

The six-method repeated-measures analyzes were significant for method, fatigue level, and the method-by-fatigue interaction under both protocols (Table 3). Holm-adjusted paired *t*-tests favored FRFNet over all five baselines at all nine fatigue levels in both protocols. These findings indicate that FRFNet provides improved unseen-subject robustness, although the lower LOSO accuracy also confirms that inter-subject variability remains a substantial challenge.

### 4.8 Component-level ablation analysis

**Figure 10:**
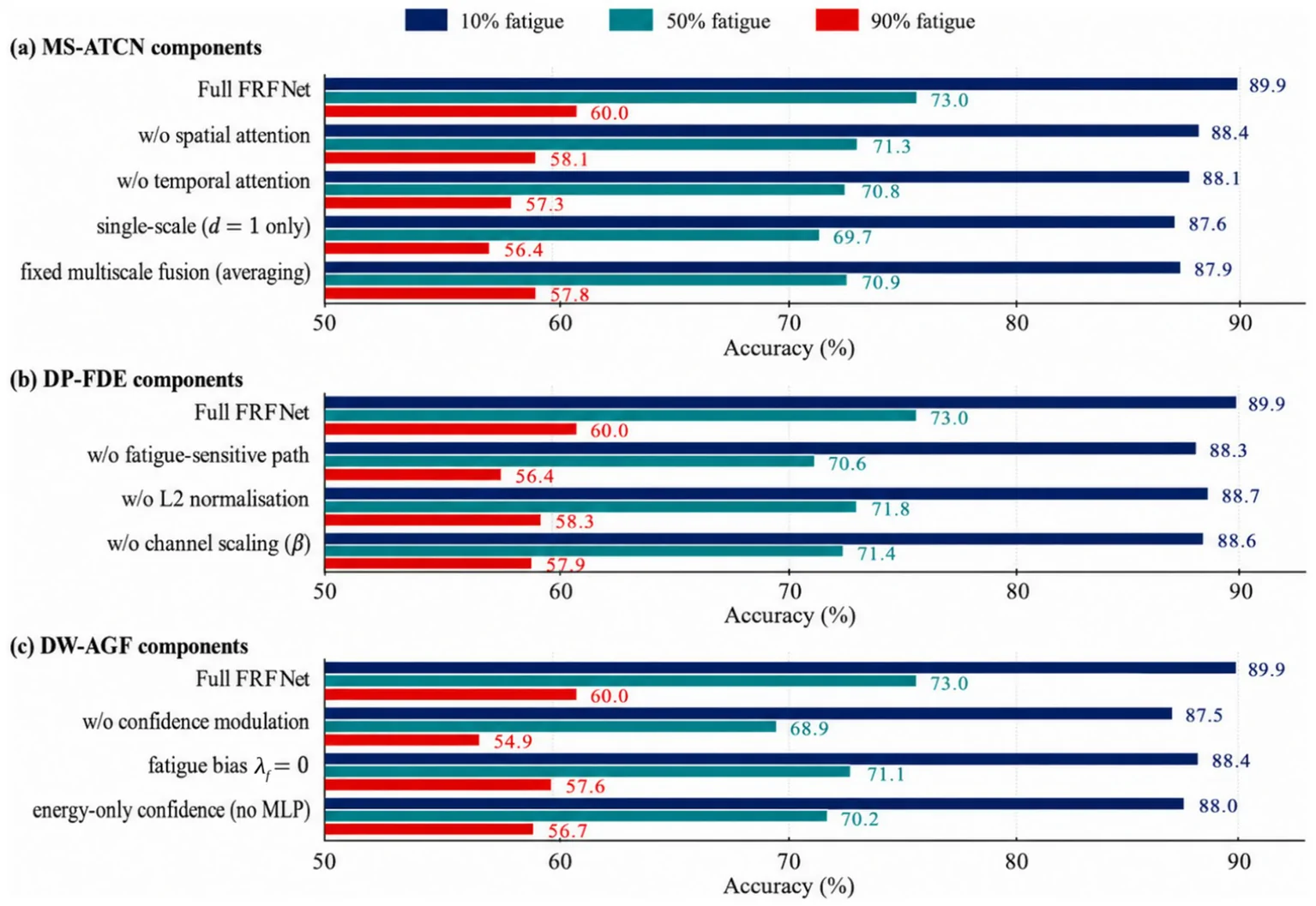
Fine-grained component-level ablation results for **(a)** MS-ATCN, **(b)** DP-FDE, and **(c)** DW-AGF at 10, 50, and 90% synthetic fatigue.

To further identify the contribution of the internal components, fine-grained ablation experiments were conducted for MS-ATCN, DP-FDE, and DW-AGF at three representative fatigue levels (10, 50, and 90%). The results are summarized in Table 4 and Figure 10. Across the tested configurations, removing individual components produced consistent numerical reductions in classification accuracy, although the magnitude of the reduction differed across components and fatigue conditions.

**Table 4:** Component-level ablation accuracy (%) at representative synthetic fatigue levels.

Variant	10% fatigue	50% fatigue	90% fatigue
**Full FRFNet**	**89.9**	**73.0**	**60.0**
MS-ATCN components			
w/o spatial attention	88.4	71.3	58.1
w/o temporal attention	88.1	70.8	57.3
single-scale (*d* = 1 only)	87.6	69.7	56.4
fixed multiscale fusion (averaging)	87.9	70.9	57.8
DP-FDE components			
w/o fatigue-sensitive path	88.3	70.6	56.4
w/o L2 normalization	88.7	71.8	58.3
w/o channel scaling (*β*)	88.6	71.4	57.9
DW-AGF components			
w/o confidence modulation	87.5	68.9	54.9
fatigue bias *λ_f_* = 0	88.4	71.1	57.6
energy-only confidence (no MLP)	88.0	70.2	56.7

Values are means across 25 participants. The full FRFNet values are included as the reference configuration. Bold values indicate the full (complete) FRFNet configuration, provided as the reference for comparison with each ablated variant.

Within MS-ATCN, replacing the multiscale encoder with a single-scale branch produced the largest decrease, reducing accuracy from 60.0 to 56.4% at 90% fatigue. Removing temporal attention reduced accuracy to 57.3%, whereas removing spatial attention resulted in 58.1%. Fixed averaging of the three temporal scales reduced accuracy to 57.8%. These results indicate that multiscale temporal modeling and sample-adaptive scale selection are the main contributors of the EEG encoder, while spatial and temporal reweighting provide additional improvements.

Within DP-FDE, removing the fatigue-sensitive path reduced accuracy to 56.4% at 90% fatigue, which was a larger decrease than removing L2 normalization (58.3%) or channel scaling (57.9%). This supports the use of a separate degradation-sensitive representation to provide fatigue-related guidance to the fusion module rather than encoding action and degradation information through a single EMG pathway.

Within DW-AGF, removing confidence modulation caused the largest overall decrease, from 60.0 to 54.9% at 90% fatigue. Setting the fatigue-bias coefficient to zero reduced accuracy to 57.6%, and replacing the learned confidence estimator with an energy-only strategy reduced it to 56.7%. Thus, both learned modality confidence and explicit degradation conditioning contribute to reliable modality reallocation under severe EMG degradation.

### 4.9 Hyperparameter sensitivity analysis

The sensitivity of FRFNet to five key hyperparameter groups was evaluated at 50% synthetic fatigue using a one-factor-at-a-time strategy. The tested factors included the dilation schedule, fatigue-bias coefficient λ_f_, confidence-regularization weight λ_q_, confidence-estimation hidden dimension, and temporal kernel size of the fusion decoder.

**Figure 11:**
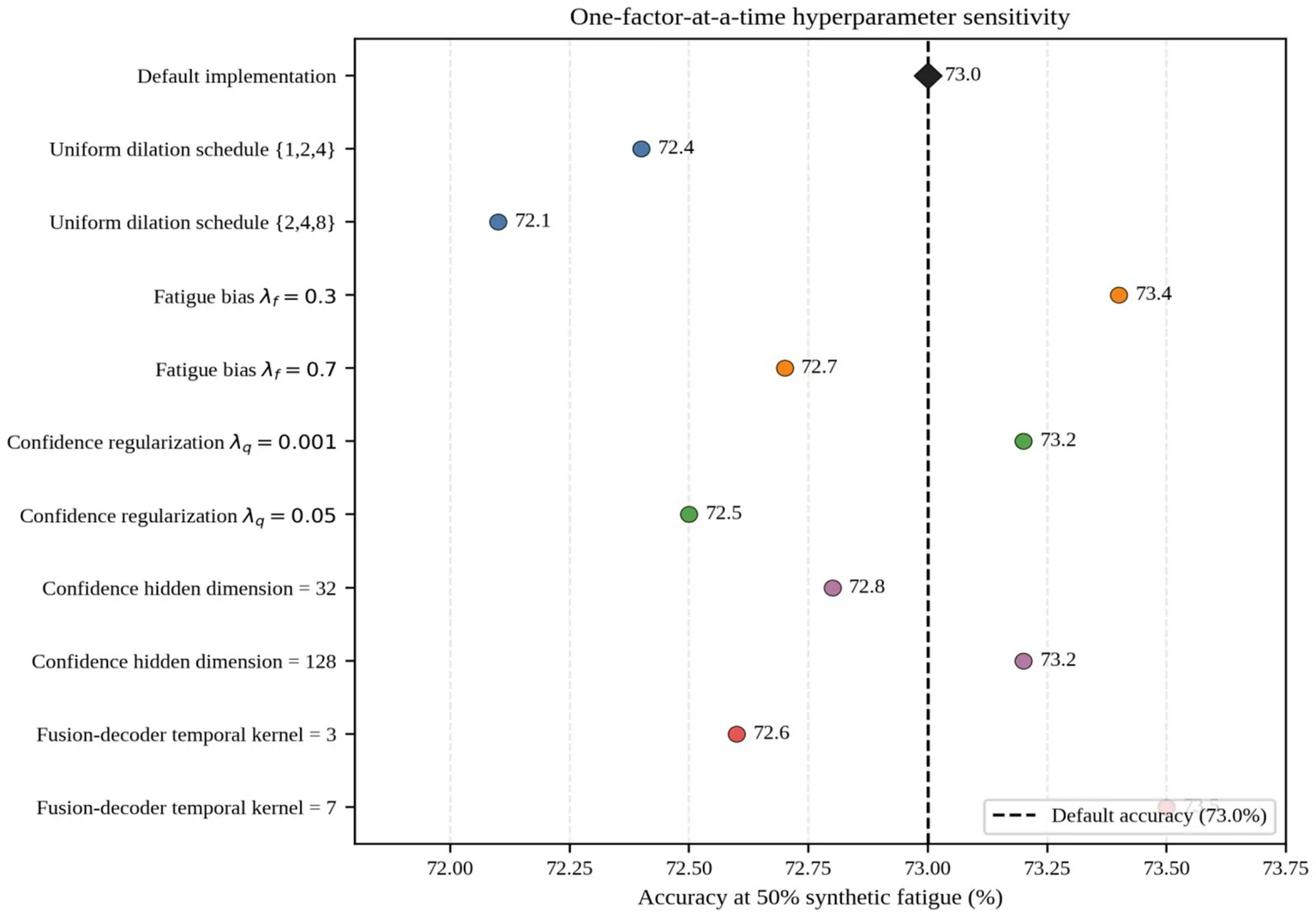
One-factor-at-a-time sensitivity of FRFNet at 50% synthetic fatigue. The dashed line denotes the default accuracy of 73.0%.

As shown in Table 5 and Figure 11, the tested accuracies ranged from 72.1 to 73.5%, whereas the default implementation achieved 73.0%. All perturbations remained within −0.9 to +0.5 percentage points of the default result. Although several alternatives produced small numerical improvements, the default setting was retained because it provided near-optimal performance with moderate representation dimension and kernel size. Performance remained within a narrow numerical range under the tested one-factor perturbations.

**Table 5:** Sensitivity of FRFNet accuracy at 50% synthetic fatigue.

Factor	Configuration	Accuracy (%)	Difference vs. default
**Default**	**Original implementation**	**73.0**	**0.0**
Dilation schedule	Uniform {1, 2, 4}	72.4	−0.6
	Uniform {2, 4, 8}	72.1	−0.9
Fatigue bias *λ_f_*	0.3	73.4	+0.4
	0.7	72.7	−0.3
Confidence regularization *λ_q_*	0.001	73.2	+0.2
	0.05	72.5	−0.5
Confidence hidden dimension	32	72.8	−0.2
	128	73.2	+0.2
Fusion-decoder temporal kernel	3	72.6	−0.4
	7	73.5	+0.5

The default implementation uses branch-specific dilation schedules: short {1, 2, 4}, medium {2, 4, 8}, and long {4, 8, 16}. Each alternative changes one factor while keeping all remaining settings fixed. Bold values indicate the default hyperparameter configuration used in the main experiments, provided as the reference for comparison with each tested alternative.

### 4.10 Computational complexity and inference efficiency

Table 6 summarizes the deployment-oriented complexity and forward-pass efficiency of the evaluated deep models. FRFNet contained 1.1651 million trainable parameters, required 3.8834 GFLOPs for the complete deployment inference path, and occupied 4.54 MB as a serialized state dictionary. Its mean single-trial forward latency was 5.321 ± 0.934 ms, with a 95th-percentile latency of 7.340 ms and a peak inference memory of 42.58 MB. E2FNet, DMEFNet-adapted, FAConformer-style, and DBF required 0.0529, 0.1129, 0.0903, and 0.0054 GFLOPs, respectively. Their mean forward latencies were 1.383, 2.388, 4.469, and 1.985 ms, respectively.

**Table 6:** Computational complexity and forward-pass efficiency under the unified deployment benchmark.

Model	Parameters (M)	FLOPs (G)	Model size (MB)	Latency (ms)	P95 latency (ms)	Peak memory (MB)
**FRFNet**	**1.1651**	**3.8834**	**4.54**	**5.321 ± 0.934**	**7.340**	**42.58**
E2FNet	0.2184	0.0529	0.87	1.383 ± 0.309	1.850	19.22
DCA Fusion	–	–	–	–	–	–
DMEFNet-adapted	0.2966	0.1129	1.16	2.388 ± 0.179	2.733	17.90
FAConformer-style	0.5670	0.0903	2.22	4.469 ± 0.633	5.805	14.53
DBF	0.0545	0.0054	0.23	1.985 ± 0.444	2.472	9.60

All neural models were tested on the same NVIDIA GeForce RTX 4060 Laptop GPU using FP32 inputs, batch size 1, 30 warm-up passes, and 200 timed forward passes. Latency covers network forward inference only. FLOPs were recomputed from the exact model implementations over the complete deployment inference path using the same input dimensions for all neural models. DCA Fusion is a non-neural handcrafted-feature and linear-classification pipeline; neural-network parameter count and forward-pass FLOPs are therefore not applicable. Bold values indicate the proposed FRFNet model, provided as the reference for comparison with the baseline methods.

FRFNet therefore introduced higher computational cost than the lightweight comparison models, reflecting its multiscale encoders and degradation-aware adaptive fusion operations. Nevertheless, the model-forward computation remained in the millisecond range. These measurements support computational feasibility at the network-inference level, but they do not constitute online or closed-loop validation because acquisition, preprocessing, communication, and control delays were not included.

### 4.11 Channel-wise EEG and EMG contribution analysis

**Figure 12:**
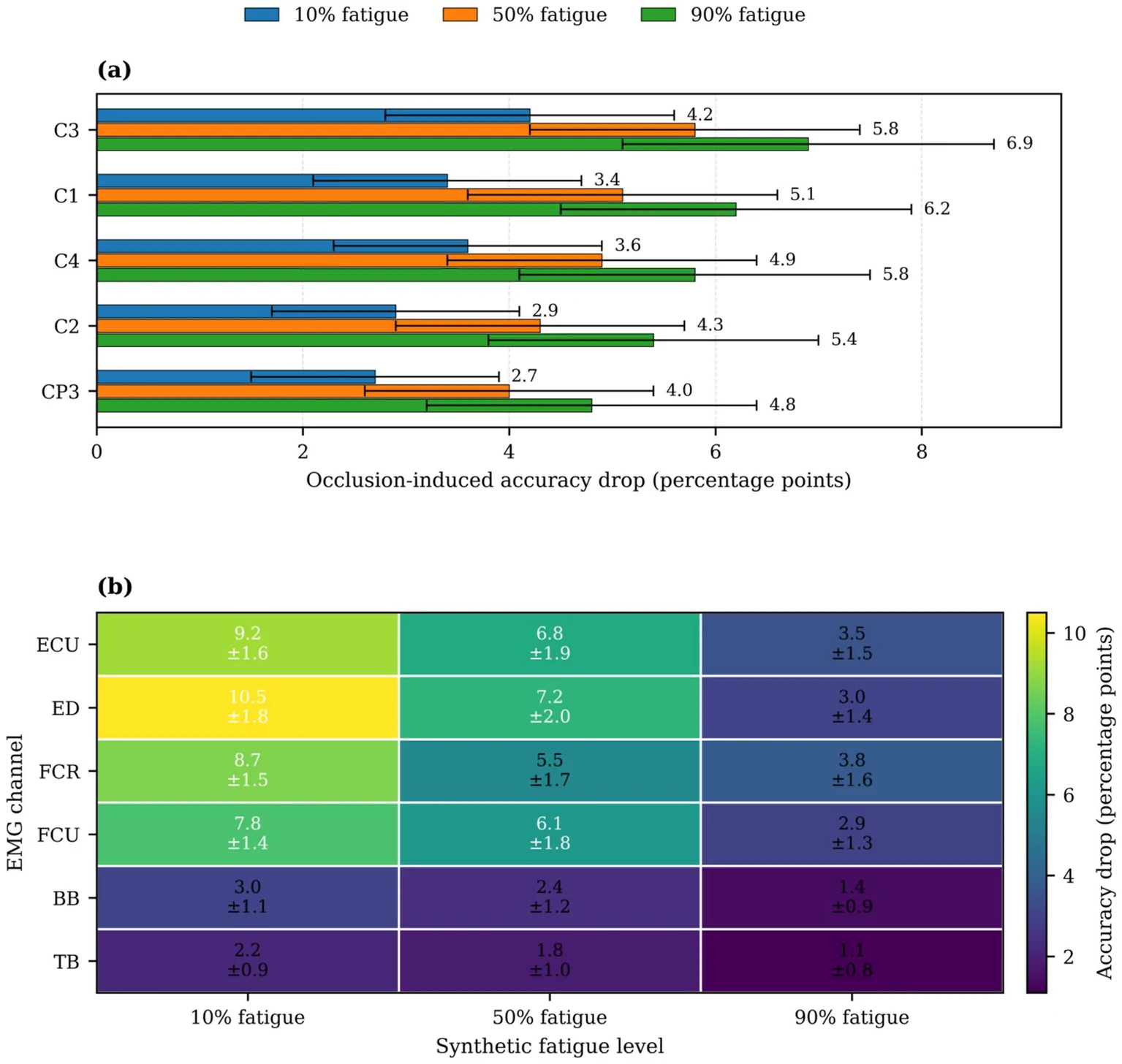
Channel-wise contribution analysis. **(a)** Top-five EEG channels ranked by the occlusion-induced accuracy reduction at 90% synthetic fatigue. Bars show the mean across 25 participants, and error bars indicate ±1 SD. **(b)** EMG channel-contribution heatmap at 10, 50, and 90% synthetic fatigue. Larger values indicate a greater reduction in accuracy after channel occlusion.

The channel-wise occlusion analysis revealed that central sensorimotor EEG electrodes made the largest predictive contributions to fatigue-robust hand movement decoding. Figure 12A presents the five EEG channels with the largest occlusion-induced accuracy reductions at 90% fatigue, with the corresponding values summarized in Table 7.

**Table 7:** Top-five EEG channels ranked by the occlusion-induced accuracy reduction at 90% synthetic fatigue.

Rank	Channel	10% fatigue Δ*Acc* (percentage points)	50% fatigue Δ*Acc* (percentage points)	90% fatigue Δ*Acc* (percentage points)
1	C3	4.2 ± 1.4	5.8 ± 1.6	6.9 ± 1.8
2	C1	3.4 ± 1.3	5.1 ± 1.5	6.2 ± 1.7
3	C4	3.6 ± 1.3	4.9 ± 1.5	5.8 ± 1.7
4	C2	2.9 ± 1.2	4.3 ± 1.4	5.4 ± 1.6
5	CP3	2.7 ± 1.2	4.0 ± 1.4	4.8 ± 1.6

Values are mean ± sample standard deviation across 25 participants under the within-subject nested cross-validation protocol. Δ*Acc* is the full-input accuracy minus the accuracy obtained after independently occluding the indicated channel; larger positive values indicate greater predictive contribution.

At 10% fatigue, occluding C3 reduced classification accuracy by 4.2 ± 1.4 percentage points. Its contribution increased to 5.8 ± 1.6 percentage points at 50% fatigue and 6.9 ± 1.8 percentage points at 90% fatigue. Similar progressive increases were observed for C1, C4, C2, and CP3. At 90% fatigue, occluding C3, C1, and C4 reduced accuracy by 6.9, 6.2, and 5.8 percentage points, respectively.

These findings indicate that FRFNet increasingly relies on central EEG information when the peripheral EMG modality becomes less reliable. The larger contribution of C3 than C4 under severe degradation is consistent with the relevance of contralateral sensorimotor activity during right-hand movement. However, the occlusion results quantify model dependence on the input channels and should not be interpreted as direct evidence of causal cortical involvement.

Figure 12B presents the EMG channel-contribution heatmap. Under low-fatigue conditions, the four forearm channels (ECU, ED, FCR, and FCU) exhibited substantially larger contributions than the upper-arm channels (BB and TB). ED produced the largest accuracy reduction at 10% fatigue, with an occlusion-induced decrease of 10.5 ± 1.8 percentage points.

As synthetic fatigue increased, the contributions of all EMG channels decreased. The contribution of ED declined from 10.5 ± 1.8 percentage points at 10% fatigue to 3.0 ± 1.4 percentage points at 90% fatigue, whereas the contribution of BB declined from 3.0 ± 1.1 to 1.4 ± 0.9 percentage points. These results indicate that the discriminative information provided by the forearm EMG channels was more strongly affected by the applied synthetic degradation protocol. Because the fatigue perturbation was synthetically generated, the results describe model reliance under signal degradation rather than direct muscle-specific physiological fatigue severity.

### 4.12 Comparison with alternative fusion strategies

**Figure 13:**
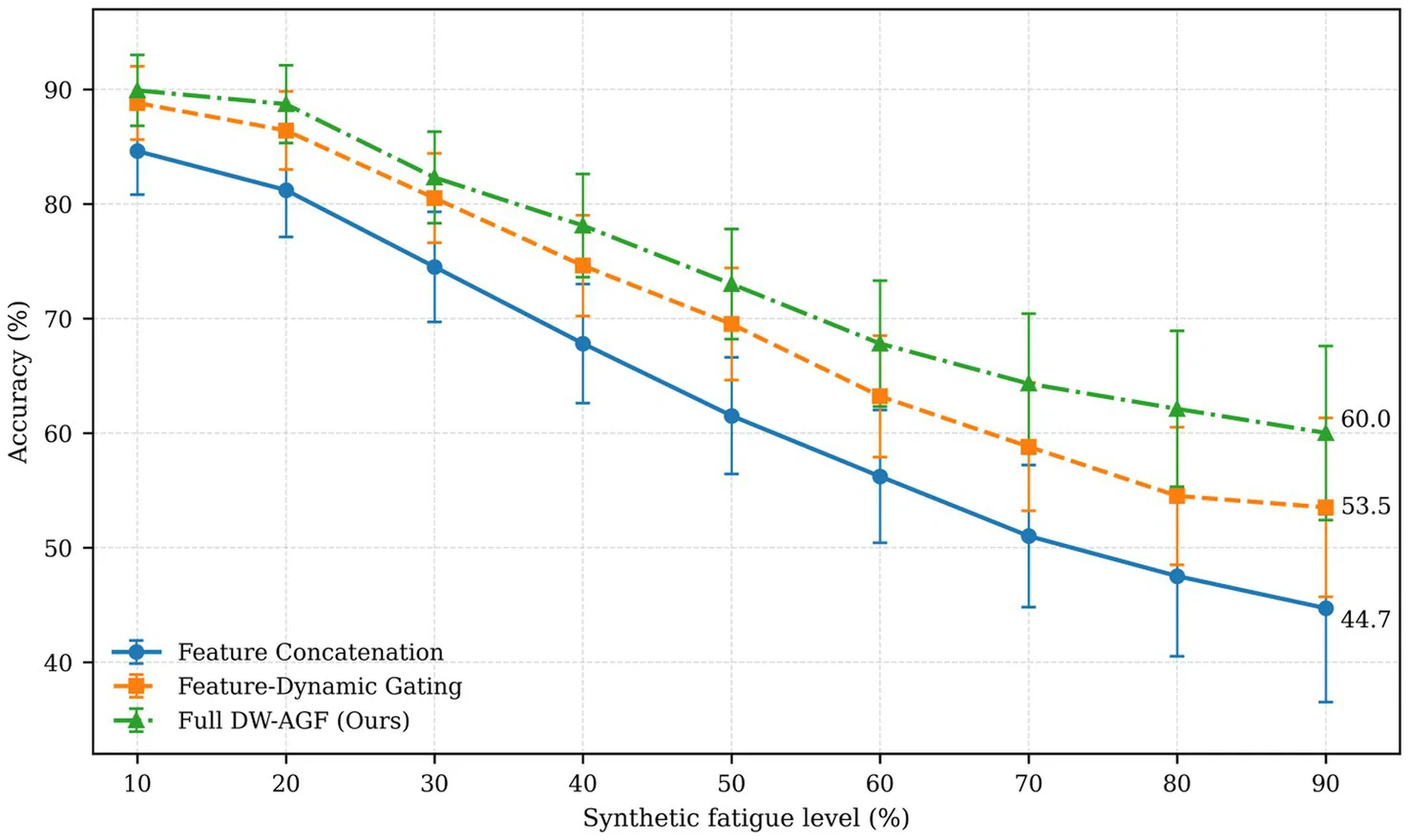
Accuracy of three representative fusion strategies across the 10–90% synthetic fatigue range. Points represent mean accuracy across 25 participants, and error bars indicate ±1 SD. The performance gap between DW-AGF and the alternative strategies widens as EMG degradation increases.

Figure 13 compares the degradation trajectories of the three fusion strategies across the complete synthetic fatigue range, with representative results summarized in Table 8.

**Table 8:** Comparison of representative fusion strategies at key synthetic fatigue levels.

Fusion strategy	10% accuracy (%)	50% accuracy (%)	90% accuracy (%)	Mean accuracy (%)
Feature concatenation	84.6 ± 3.8	61.5 ± 5.1	44.7 ± 8.2	63.2
Feature-dynamic gating	88.8 ± 3.2	69.5 ± 4.9	53.5 ± 7.8	70.0
**Full DW-AGF (ours)**	**89.9 ± 3.1**	**73.0 ± 4.8**	**60.0 ± 7.6**	**74.0**

Values are mean ± sample standard deviation across 25 participants. All configurations used identical modality encoders, co-attention modules, classifiers, optimization settings, data partitions, and random seeds; only the fusion operation was changed. Mean accuracy is the arithmetic average across the nine fatigue levels from 10 to 90%. Bold values indicate the complete proposed DW-AGF fusion strategy, provided as the reference for comparison with the alternative fusion strategies.

Feature Concatenation exhibited the most pronounced performance degradation, decreasing from 84.6 ± 3.8% at 10% fatigue to 44.7 ± 8.2% at 90% fatigue. Feature-Dynamic Gating improved performance by generating sample-dependent fusion weights, but its accuracy still decreased to 53.5 ± 7.8% under severe degradation.

In contrast, the complete DW-AGF achieved 60.0 ± 7.6% accuracy at 90% fatigue, outperforming Feature Concatenation and Feature-Dynamic Gating by 15.3 and 6.5 percentage points, respectively. Averaged across the nine fatigue levels, DW-AGF achieved 74.0% accuracy, compared with 70.0% for Feature-Dynamic Gating and 63.2% for Feature Concatenation.

The performance gap between DW-AGF and Feature-Dynamic Gating increased from 1.1 percentage points at 10% fatigue to 3.5 percentage points at 50% fatigue and 6.5 percentage points at 90% fatigue. This widening gap indicates that feature-dependent gating alone becomes insufficient when EMG degradation is severe. The explicit fatigue estimate and modality-confidence information provide additional context for reliability-aware modality reallocation.

## 5 Discussion

### 5.1 Effectiveness of fatigue-aware adaptive fusion

The results demonstrate that fatigue-aware adaptive fusion is effective for EEG–EMG movement intention recognition under modality degradation. Conventional fusion models often learn a fixed or implicit fusion pattern from training data. When training data mainly represent normal or low-fatigue conditions, the model may rely heavily on EMG because EMG is highly discriminative for executed hand movements. However, fatigue reduces EMG reliability, and degraded EMG features may then dominate the fused representation.

FRFNet addresses this problem through fatigue estimation and dynamic modality weighting. The fatigue-sensitive EMG path estimates degradation from energy attenuation, while the confidence module evaluates modality quality. These signals guide DW-AGF to shift the fusion strategy from EMG-dominant under low fatigue to EEG-dominant under severe fatigue. This gradual transition prevents abrupt modality switching and explains why FRFNet maintains higher accuracy than conventional fusion methods under moderate and severe fatigue.

### 5.2 Roles of EEG and EMG in fatigue-robust recognition

EEG and EMG play different roles in FRFNet. EEG provides relatively stable cortical intention information when EMG becomes unreliable, but EEG alone has limited discriminative ability for fine grasp classification. Therefore, the role of EEG is not to replace EMG completely, but to provide a stable complementary source under fatigue. The MS-ATCN module improves EEG representation by extracting multiscale temporal patterns and applying spatial–temporal attention.

EMG remains valuable under normal and mild fatigue conditions because it directly reflects muscle activation. In the experiments, EMG-only achieved high accuracy under 10 and 20% fatigue, confirming its strong action-discriminative ability. However, its performance dropped sharply under severe fatigue. DP-FDE reduces this problem by separating fatigue-invariant action features from fatigue-sensitive degradation information. Without this separation, action-related features and fatigue factors are more likely to be entangled. The ablation results confirm that both stable EEG representation and fatigue-disentangled EMG encoding contribute to the final robustness of FRFNet.

The strict LOSO results further show that FRFNet achieved the highest absolute mean accuracy among the evaluated methods when tested on unseen participants. This indicates that degradation-aware modality reallocation is useful beyond subject-specific calibration, although absolute performance remains lower than in the within-subject setting. Because the within-subject and LOSO protocols differ in subject composition and training-set size, their raw performance difference was not interpreted as a standalone generalization metric. The additional macro-F1 and *κ* results support the same overall conclusion and reduce reliance on accuracy as the sole outcome measure.

The fine-grained ablation analysis clarifies the internal sources of robustness. Confidence modulation in DW-AGF and the fatigue-sensitive path in DP-FDE produced the largest reductions when removed, particularly under severe degradation. The MS-ATCN results further indicate that complementary temporal receptive fields and learnable scale fusion are more influential than any single attention component. Together with the sensitivity analysis, these findings suggest that the performance gain arises from coordinated component interactions rather than a narrowly tuned hyperparameter setting.

The channel-wise occlusion analysis further showed that central sensorimotor EEG channels, particularly C3, C1, and C4, became increasingly important as EMG degradation progressed. This pattern supports the interpretation that FRFNet shifts its reliance toward relatively stable cortical information when peripheral signals become unreliable. In contrast, forearm EMG channels provided strong discriminative information under low-fatigue conditions but showed pronounced contribution reductions under severe synthetic degradation. These results provide channel-level interpretation of the modality-reallocation behavior. Nevertheless, occlusion importance reflects model dependence and does not establish a causal relationship between individual electrodes or muscles and physiological fatigue.

### 5.3 Comparison with existing fusion and EEG decoding methods

Compared with existing EEG decoding and EEG–EMG fusion methods, FRFNet focuses specifically on fatigue-induced modality reliability imbalance. Traditional EEG methods, such as CSP, FBCSP, spatio-spectral filtering, and regularized CSP, provide useful spatial and spectral representations but are mainly designed for single-modal EEG decoding (Ramoser et al., 2000; Ang et al., 2008; Lemm et al., 2005; Dornhege et al., 2006; Thomas et al., 2009; Lotte and Guan, 2010). Deep learning models such as Deep ConvNet, EEGNet, FBCNet, EEG Conformer, and LMDA-Net further improve EEG feature extraction through convolution, multi-view spectral representation, self-attention, and multi-dimensional attention (Schirrmeister et al., 2017; Lawhern et al., 2018; Mane et al., 2021; Song et al., 2023; Miao et al., 2023). However, these methods do not address the reliability imbalance between EEG and EMG under fatigue.

Existing EEG–EMG fusion models have demonstrated the value of multimodal biological signals. Tryon and Trejos showed that EEG–EMG fusion is feasible for dynamic task weight classification and CNN-based fusion (Tryon and Trejos, 2021a; Tryon and Trejos, 2021b). Yang et al. used functional connectivity and graph convolution to improve hand motion recognition under simulated fatigue (Yang et al., 2022). Shi et al. proposed dense co-attention for multimodal human–exoskeleton movement prediction (Shi et al., 2022). Tang et al. improved EEG–sEMG fusion decoding using attention-based multiscale parallel convolution and dual-stream global–local spectral fusion (Tang et al., 2024; Tang et al., 2025). Jiang et al. proposed E2FNet using multiscale convolution and cross-modal feature enhancement (Jiang et al., 2024). These methods enhance cross-modal interaction, but most of them do not explicitly estimate fatigue degree or dynamically suppress degraded EMG features.

The main difference is that FRFNet introduces fatigue-aware modeling into both EMG representation and modality fusion. DP-FDE separates fatigue-invariant action features from fatigue-sensitive degradation information, while DW-AGF uses modality confidence and fatigue estimation to adjust EEG and EMG contributions. Therefore, FRFNet can use EMG effectively under low fatigue and reduce its influence when fatigue becomes severe.

The fusion-strategy comparison further demonstrated that sample-dependent feature gating without explicit fatigue estimation was insufficient under severe EMG degradation. Although Feature-Dynamic Gating consistently outperformed simple feature concatenation, the increasing performance gap between Feature-Dynamic Gating and DW-AGF highlights the complementary roles of explicit fatigue conditioning and modality-confidence modulation. These components allow the fusion module to distinguish reliability degradation from ordinary feature variation and to reallocate modality contributions accordingly.

### 5.4 Practical implications and limitations

The proposed method has potential value for rehabilitation robots, wearable assistive devices, and long-duration human–machine interaction systems. During repeated rehabilitation training, users may gradually become fatigued, and fixed fusion strategies may lead to unstable decoding. FRFNet provides a fatigue-aware solution, evaluated here under synthetic EMG degradation, by adjusting modality contributions according to estimated signal reliability. This idea may also be extended to other degradation scenarios, such as electrode shift, impedance variation, and unstable muscle activation.

The computational analysis indicates that the increased representational capacity of FRFNet is accompanied by a measurable but bounded inference overhead. The model used 1.1651 million parameters and achieved a mean forward-pass latency of 5.321 ms under the stated GPU benchmark. Although the forward-pass latency of FRFNet was higher than those of the lightweight E2FNet, DMEFNet-adapted, and DBF implementations, it remained in the millisecond range, indicating that the additional computational overhead introduced by the proposed framework is limited at the network-inference level. However, because the present benchmark did not include signal acquisition, preprocessing, communication, or actuator-response delays, these measurements should not be interpreted as end-to-end real-time system latency.

This study still has limitations. The current dataset contains healthy participants and focuses on three representative grasp classes. Broader validation across participants, recording sessions, clinical populations, and movement categories will further establish the generality of the proposed framework. A prospective synchronized EEG-sEMG study incorporating standardized fatigue induction, independent physiological reference measures, cross-session acquisition, and clinical populations is therefore explicitly planned as the next stage of validation to determine whether the robustness observed under controlled degradation translates to genuine rehabilitation-related fatigue. Independent references in such a study should include both objective and subjective fatigue markers, such as decline in maximum voluntary contraction (MVC), ratings of perceived exertion (RPE), and fatigue-sensitive sEMG spectral or muscle-fiber conduction-velocity indices. In addition, the present evaluation remains offline. Although the measured network-forward latency suggests compatibility with low-latency decoding, end-to-end online latency, including signal acquisition, preprocessing, data transmission, device communication, and actuator response, was not assessed. Online adaptation and closed-loop control stability should therefore be validated in future real-time rehabilitation experiments.

## 6 Conclusion

This study proposed FRFNet, a fatigue-robust EEG–EMG fusion network for hand movement intention recognition. The model combines multiscale EEG temporal modeling, dual-path EMG fatigue disentanglement, enhanced co-attention interaction, and fatigue-aware dynamic weighted adaptive gating. The EEG branch extracts stable cortical movement intention features, while the EMG branch separates action-related fatigue-invariant features from fatigue-sensitive degradation information. The dynamic gating module adaptively adjusts the contribution of EEG and EMG according to modality confidence and fatigue estimation.

Experiments on a public multimodal EEG–EMG dataset showed that FRFNet maintained better recognition performance than the evaluated baselines under synthetic fatigue-related EMG degradation. The advantage remained under strict LOSO testing, although all methods exhibited lower unseen-subject performance. Subject-level statistical analyzes, additional multiclass metrics, core-module ablations, component-level ablations, and hyperparameter sensitivity analyzes consistently supported the contribution of degradation-aware representation and confidence-guided dynamic fusion. Channel-wise occlusion and alternative-fusion comparisons further supported the electrode-level, muscle-level, and fusion-mechanism interpretations of the proposed framework. The deployment benchmark further showed that FRFNet performed single-trial network inference in 5.321 ± 0.934 ms on the evaluated GPU, supporting low-latency computational feasibility at the model-forward level.

Overall, FRFNet provides an effective framework for EEG–EMG fusion under controlled modality degradation. The results demonstrate the value of degradation-aware representation learning and reliability-guided modality reallocation for robust movement-intention decoding in long-duration rehabilitation and adaptive human–machine interaction. Future work will validate these findings in a prospective synchronized EEG-sEMG study with standardized fatigue induction, independent physiological references, cross-session acquisition, and clinical populations.

## Data Availability

Publicly available datasets were analyzed in this study. The original multimodal EEG–EMG dataset can be found at: https://doi.org/10.5524/100788.
